# Integrated Analysis of Crucial Genes and miRNAs Associated with Osteoporotic Fracture of Type 2 Diabetes

**DOI:** 10.1155/2022/3921570

**Published:** 2022-08-10

**Authors:** Liang Mo, Zhangzheng Wang, Haoran Huang, Jianxiong Li, Chao Ma, Jiahao Zhang, Fayi Huang, Wei He, Yuhao Liu, Chi Zhou

**Affiliations:** ^1^The First Affiliated Hospital of Guangzhou University of Chinese Medicine, Guangzhou, 510405 Guangdong Province, China; ^2^Guangdong Provincial Hospital of Traditional Chinese Medicine, Guangzhou, 51000 Guangdong Province, China

## Abstract

**Purpose:**

The aim of this study is to explore pathological mechanisms of bone fragility in type 2 diabetes mellitus (T2DM) patients.

**Methods:**

Identifying common genes for T2DM and osteoporosis by taking the intersection is shared by the Comparative Toxicogenomics Database (CTD), DISEASES, and GeneCards databases. The differentially expressed genes (DEGs) and the differentially expressed miRNAs (DEMs) were identified by analyzing the Gene Expression Omnibus (GEO) datasets (GSE35958, GSE43950, and GSE70318). FunRich and miRNet were applied to predict potential upstream transcription factors and downstream target genes of candidate DEMs, respectively. The Gene Ontology (GO) and Kyoto Encyclopedia of Genes and Genomes (KEGG) enrichment analyses were performed to explore potential mechanisms using Metascape. Eventually, a miRNA-gene network was constructed by Cytoscape software.

**Results:**

271 common targets and 35 common DEGs between T2DM and osteoporosis were screened out in the above databases, and a total of ten DEMs were obtained in the GSE70318. SP1 was predicted to potentially regulate most of the DEMs. Enrichment analysis showed the PI3K-Akt signaling pathway and AGE-RAGE signaling pathway in diabetic complications may play an important role in diabetic skeletal fragility. Two genes (NAMPT and IGFBP5) were considered as key genes involving in the development of diabetic osteoporosis. Through the construction of the miRNA-gene network, most of the hub genes were found to be potentially modulated by miR-96-5p and miR-7-5p.

**Conclusion:**

The study uncovered several important genes, miRNAs, and pathological mechanisms involved in diabetic skeletal fragility, among which the PI3K-Akt signaling pathway and AGE-RAGE signaling pathway in diabetic complications may play important roles.

## 1. Introduction

Type 2 diabetes mellitus (T2DM) is a common endocrine metabolic disease in humans and accounts for more than 90% of all patients with diabetes [1]. Accumulating studies had shown that T2DM was relevant to a lot of chronic conditions, including coronary artery disease, kidney disease, diabetic retinopathy, and neuropathy, as well as bone disorders [2]. A recent study indicated that 37.8% of Chinese diabetic patients suffered from osteoporosis [3]. In addition, multiple studies have demonstrated that T2DM was suggested as an independent risk factor for osteoporotic fractures [4, 5], and the risk of having a fragility fracture in T2DM patients increased 1-3 fold compared to healthy controls [6]. Osteoporosis-associated fracture has been taken into account as an important complication of T2DM [7]. Previous studies have shown that T2DM negatively affects bone strength regardless of bone mineral density [8, 9]. Farr and Khosla [10] found that there were quality defects in both cortical and trabecular bones in T2DM patients. Moreover, bone microindentation testing displayed lower bone material strength in T2DM patients compared to those without diabetes [11]. Histomorphometric analysis of bone also showed that bone trabecular and cortical microarchitecture are both deranged in T2DM patients and may contribute to bone fragility [12]. However, the more exact molecular basis between skeleton fragility and T2DM is still not fully understood.

Several factors including obesity, hyperinsulinemia, hyperglycemia, accumulation of advanced glycosylation end products (AGEs), and presence of microvascular disease were considered being involving the pathogenesis of diabetic skeletal fragility [6]. Obesity and hyperinsulinemia typically emerge in the early phase of diabetes, and they can induce bone resorption by stimulating osteoclast activity through promoting a chronic inflammation environment [13]. AGEs, producing by nonenzymatic glycation of various proteins, can mediate generation of reactive oxygen species (ROS) and inflammatory cytokines, thus inducing osteoclastogenesis and osteoblast dysfunction [14]. Furthermore, the Wnt/*β*-catenin pathway has been shown to negatively regulate bone formation in T2DM patients [15]. Sclerostin is an important regulator of the Wnt/*β*-catenin pathway. Studies showed that patients with T2DM have higher serum levels of sclerostin, which can bind to Wnt coreceptors, inhibiting osteoblastogenesis and bone formation [16, 17]. These findings strongly suggested the interaction between bone metabolism impairment and glucose metabolism. However, they were mainly from clinical perspectives, and few studies had investigated genomic relationship between them.

**Figure 1:**
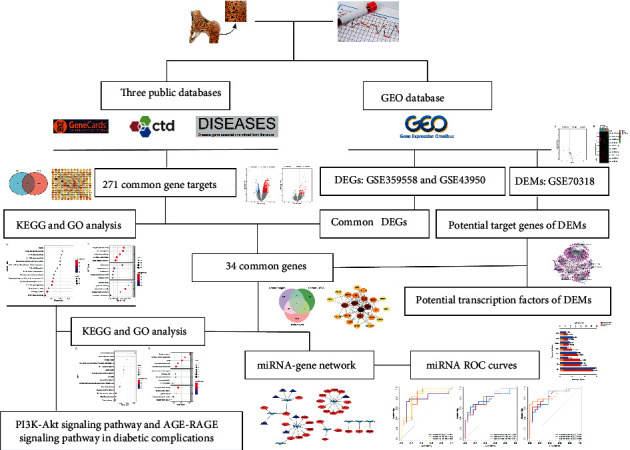
Schematic diagram of the study design.

In this study, we explored the common pathogenesis between T2DM and osteoporosis by combining the multisource T2DM and osteoporosis-related data. Simultaneously, we constructed a miRNA-gene network to identify some potential miRNAs and genes involved in T2DM osteoporotic fracture. Our study offered a new approach to identify pathological mechanisms and potential targets for T2DM with osteoporotic fracture. The research workflow is shown in Figure 1.

## 2. Methods

### 2.1. Data Gathering

Data in this study were obtained from public databases and gene expression databases. The Comparative Toxicogenomics Database (CTD) is a publicly available database which provides information about interactions between environmental chemicals and gene products and their effect on human diseases [18]. The GeneCards is a searchable, comprehensive database that provides comprehensive information on human genetic information comprehensively. The knowledge base automatically integrates gene-centric data from more than 100 web sources, including genomic, transcriptomic, proteomic, genetic, clinical, and functional information [19]. The DISEASES database incorporates evidence such as the Genetics Home Reference, UniProtKB, and DistiLD [20]. We extracted data related with T2DM and osteoporosis from the CTD (https://ctdbase.org/), GeneCards (/http://www.genecards.org/), and DISEASES (https://diseases.jensenlab.org/) that were downloaded in October 2021.We selected the “type 2 diabetes mellitus” and “osteoporosis” as the keywords.

### 2.2. Common Gene Targets between T2DM and Osteoporosis

The common disease-related genes between T2DM and osteoporosis from these three databases were obtained using Venn diagram, which was drawn by the website (https://www.bioinformatics.com.cn), a free online platform for data analysis and visualization. All these genes were considered to play an important role in the common pathogenesis between T2DM and osteoporosis and were extracted for further analysis.

### 2.3. Enrichment Analysis and PPI Network Construction

The Metascape [21] (https://metascape.org/), an excellent integrated analytics platform that combines functional enrichment, interactome analysis, and gene annotation within one integrated porta, was used to perform the functional analysis. The reviewed items include the Kyoto Encyclopedia of Genes and Genomes (KEGG), GO cellular component, GO biological process, and GO molecular function. Min overlap = 3 and Min enrichment = 1.5 were the screening conditions. *P* < 0.01 was considered statistically significant.

The protein-protein interaction (PPI) network was analyzed using the String database (https://string-db.org). An interaction with a corresponding combined score was selected and used to construct a PPI network with Cytoscape software. Cytoscape (version 3.7.2) is an open-source software that creates and surveys the molecular interaction network [22].

### 2.4. Analysis of Datasets in GEO

Gene expression profiles were collected from the Gene Expression Omnibus (GEO, http://www.ncbi.nlm.nih.gov/geo/) by searching the “type 2 diabetes mellitus” and “osteoporosis” terms. Eventually, the three microarray datasets (GSE35958 [23], GSE43950 [24], and GSE70318 [25]) were downloaded from the GEO database. The GSE35958 dataset contains five samples of human mesenchymal stem cells from osteoporosis patients and the other four bone marrows from nonosteoporotic age-matched donors after total hip arthroplasty. The GSE43950 includes a total of 14 samples: nine type 2 diabetes CD34+ cells samples and five age-matched healthy CD34+ cells samples in peripheral blood. The GSE7031 dataset includes serum microRNA information from postmenopausal women. Furthermore, T2DM without osteoporotic fractures was selected as the control group (*n* = 19) and T2DM with osteoporotic fractures was selected as the experimental group (*n* = 19). The platforms of the GSE35958, GSE43950, and GSE70318 were GPL570, GPL10379, and GPL20631, respectively.

GEO2R, an interactive web tool, was used to screen out differentially expressed miRNAs (DEMs) and differentially expressed genes (DEGs) between the experimental group and control group. |logFC| > 1 and the adjusted *P* value < 0.05 were considered to indicate statistical significance. To show the differential expression of DEMs and DEGs in different samples, the plot and heat map packages in the R studio were applied to draw the volcano map and heat map.

### 2.5. Target Gene Prediction of DEMs

To identify the regulatory mechanisms and functions of miRNAs, the miRNet (https://www.mirnet.ca/), a comprehensive atlas of miRNA-target interactions that contains information about miRNA-target interactions resulting from the existing miRNA-target prediction programs (such as TarBase, miRTarBase, miRecords, and miRanda) and displays the association in a visual network, was applied to predict the potential target genes of candidate DEMs.

### 2.6. Prediction of Upstream Transcription Factors of DEMs

FunRich (http://www.funrich.org/), an independent software tool mainly for functional enrichment and interaction network analysis of genes and proteins, was used to predict the potential upstream transcription factors. The *P* value < 0.05 was considered statistically significant.

### 2.7. Integrated Analysis and Construction of the miRNA-Gene Interaction Network

The common disease gene targets between T2DM and osteoporosis in the three public databases, the common DEGs, and the target genes of the DEMs were used to find the overlapping targets by using Venn diagram. These overlapping genes were considered as hub genes and suggested to function in inducing osteoporotic fractures in T2DM patients.

Overlapping genes obtained by the above methods were further used to construct the miRNA-gene interaction network by putting the miRNAs-gene pairs selected above together, and Cytoscape software (version 3.7.2) [22] was used to visualize it simultaneously.

### 2.8. ROC Curve Analysis

Receiver operating characteristic (ROC) curve analysis, a commonly used method to determine the performance of diagnostic biomarkers, is widely used in biostatistics. Herein, ROC curves were constructed to discriminate T2DM with the osteoporotic fracture group from the control T2DM group for the serum miRNAs, and the areas under the ROC curves (AUCs) were analyzed to measure the diagnostic accuracy of each identified miRNA in the miRNA-gene interaction network. The ROC plot was calculated and visualized using GraphPad Prism v5.0, and the AUC value was used to evaluate the ROC curve, with values between 0 (lowest) and 1 (highest) performance.

## 3. Results

### 3.1. Identification of Common Targets from the Three Databases

**Figure 2:**
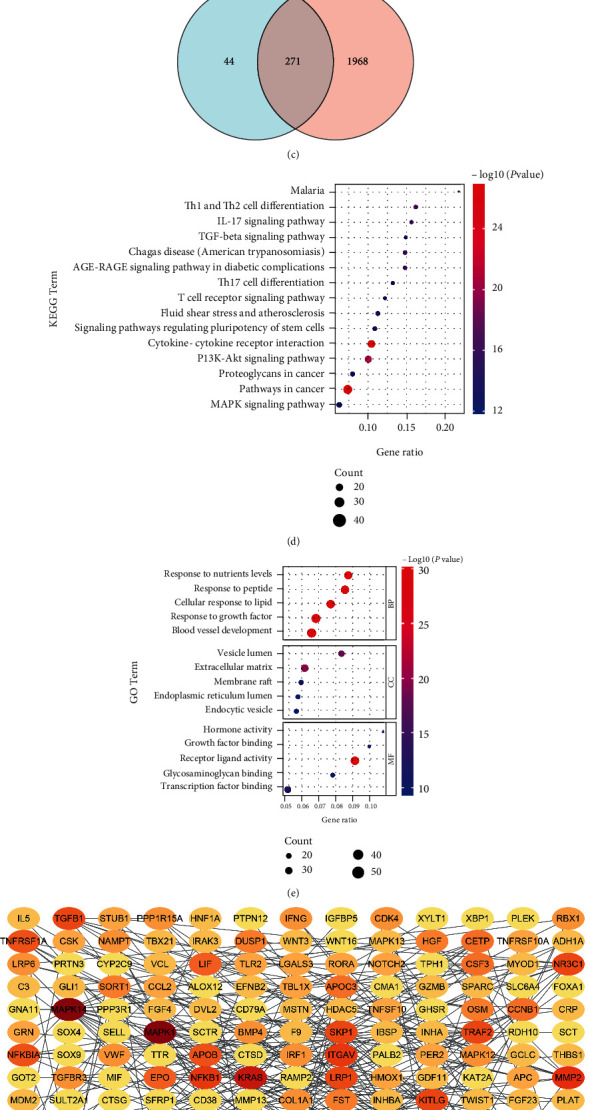
The analysis of two disease gene targets in the three public databases. (a–c) Venn diagram of intersection between T2DM- and osteoporosis-related gene targets. (d) KEGG pathway analysis results of 271 common gene targets. (e) GO analysis results of 271 common gene targets. (f) PPI network diagram of 271 common gene targets with a combined score > 0.9. OP: osteoporosis; T2DM: type 2 diabetes mellitus.

To integrate the disease-related biological data, T2DM-related genes and osteoporosis-related genes available in the GeneCards, DISEASES, and CTD databases were combined. Finally, 271 genes were determined to be shared among the three resources and the detailed information is listed in Supplemental Table S1. The Venn diagram of intersection between T2DM- and osteoporosis-related gene targets is depicted in Figures 2(a), 2(b), and 2(c).

### 3.2. Enrichment Analysis and PPI Network of Common Targets from the Three Databases

Then, the GO and KEGG analyses on the 271common targets were performed using the Metascape database.  Figure 2(d) contains the top 15 results of KEGG analysis, which consists of pathways in cancer, cytokine-cytokine receptor interaction, the P13K-Akt signaling pathway, Th1 and Th2 cell differentiation, and the AGE-RAGE signaling pathway in diabetic complications. GO biological process analysis showed that the 271 genes were particularly enriched in response to peptide, response to growth factor, blood vessel development, response to nutrient levels, and cellular response to lipid. The top five GO molecular function analysis results of the common genes are receptor ligand activity, transcription factor binding, glycosaminoglycan binding, growth factor binding, and hormone activity. As for the top five GO cellular component analysis results, we found extracellular matrix, vesicle lumen, membrane raft, endocytic vesicle, and endoplasmic reticulum lumen.  Figure 2(e) presents this information of GO functional annotation. Furtherly, the PPI network of 271 common targets (182 nodes, 305 edges) with a combined score > 0.9 was obtained by Cytoscape (Figure 2(f)).

### 3.3. Analysis of the Common DEGs

**Figure 3:**
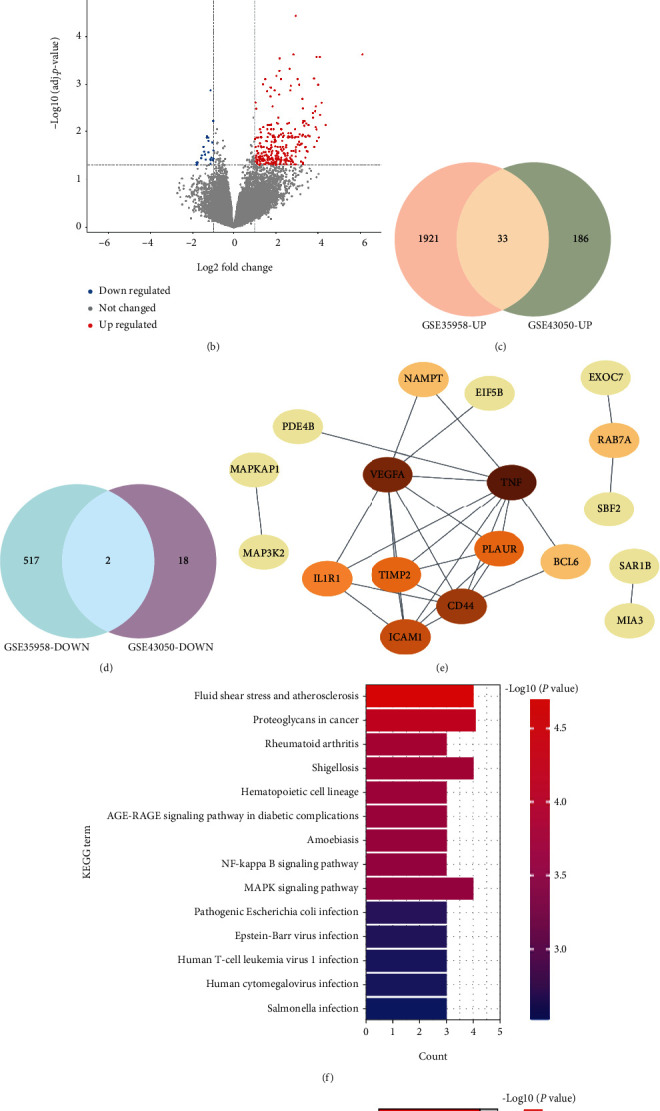
The analysis results of the common DEGs. (a) Volcano map of the GSE35958. (b) Volcano map of the GSE43950. The |logFC| > 1 and an adjusted *P* value < 0.05 were set as the threshold to screen DEMs. Red spots represent upregulated genes, and green spots represent downregulated genes in the volcano map. (c) The Venn diagram of the upregulated genes between the GSE35958 and GSE43950. (d) The Venn diagram of the downregulated genes in the GSE35958 and GSE43950. (e) PPI network diagram of the common DEGs with a combined score > 0.4. (f) KEGG pathway analysis results of common DEGs. (g) GO enrichment analysis results of common DEGs.

After analysis of GEO2R, DEGs (2473 in the GSE35958 including 1954 upregulated genes and 519 downregulated genes, and 239 in the GSE43950 including 219 upregulated and 20 downregulated genes) were identified by limiting the adjusted *P* value < 0.05 and |logFC| > 1 (Figures 3(a) and 3(b)). Among these DEGs, 33 genes were upregulated and two genes were downregulated in both the GSE35958 and GSE43950, which are shown in Figure 3(c) and Figure 3(d). The detailed information about 35 common DEGs is listed in Table 1. The PPI network of these 35 common genes with a combined score > 0.4 is shown in Figure 3(e). The KEGG and GO enrichment analyses were also performed by Metascape. KEGG analysis results showed that these genes were mainly enriched in fluid shear stress and atherosclerosis, rheumatoid arthritis, the AGE-RAGE signaling pathway in diabetic complications, the NF-kappa B signaling pathway, and the MAPK signaling pathway (Figure 3(f)). In terms of GO enrichment analysis, they were mainly involved in regulation of cell-cell adhesion, positive regulation of leukocyte migration, secretory granule membrane, and cytokine activity (Figure 3).

### 3.4. Identification of DEMs and Their Predicted Targets

**Figure 4:**
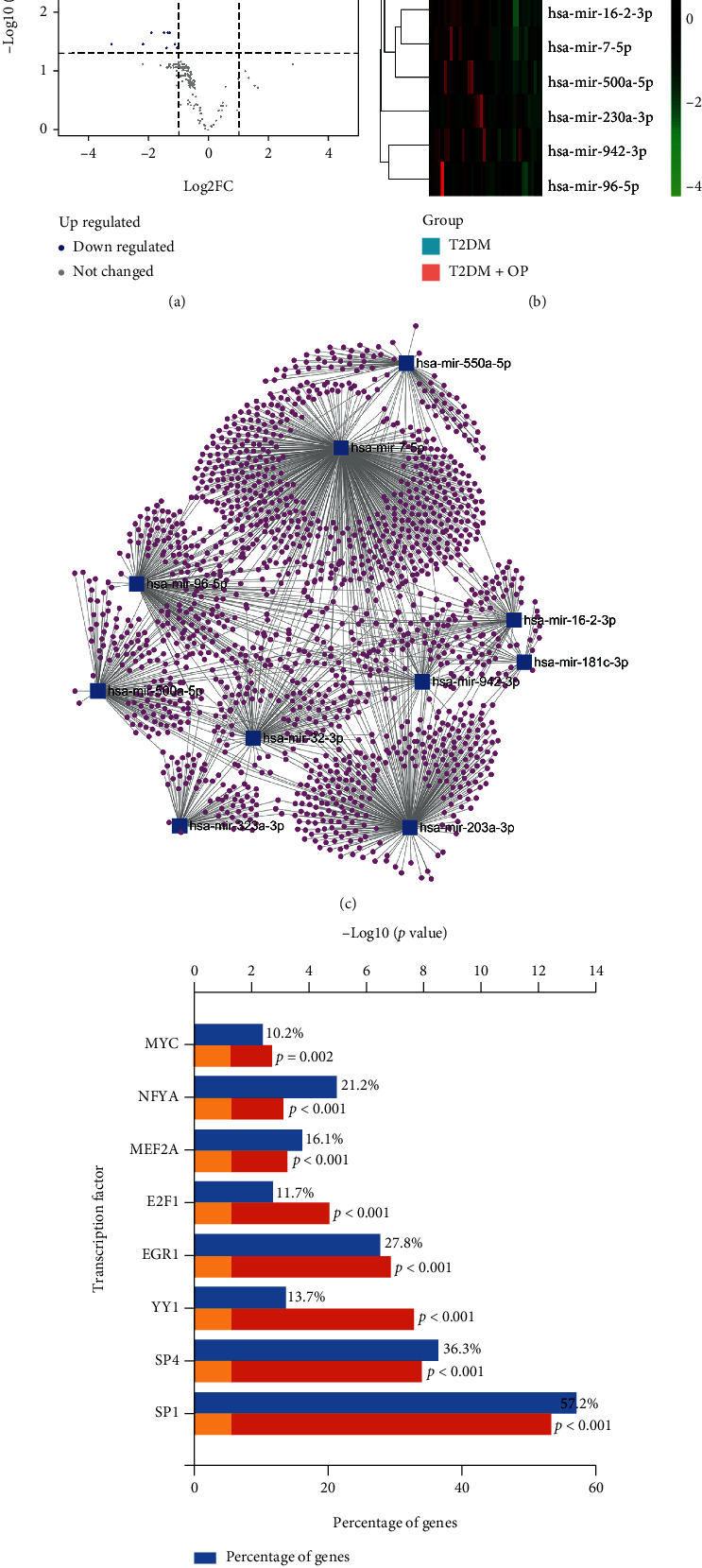
The analysis results of the GSE70318 dataset. (a) Volcano map of the GSE70318. (b) Heat map of the GSE70318. The |logFC| > 1 and an adjusted *P* value < 0.05 were set as the threshold to screen DEMs. Green spots represent downregulated miRNAs in the volcano map; red color represents high expression and blue color represents low expression in the heat map. (c) Potential target genes of DEMs predicted by miRNet. (d) Potential transcription factors of DEMs predicted by FunRich. OP: osteoporosis; T2DM: type 2 diabetes mellitus.

After screening with the threshold of an adjusted *P* value < 0.05 and |logFC| > 1, ten DEMs were identified in the GSE70318, and they are all downregulated DEMs including miR-550a-5p, miR-500a-5p, miR-181c-3p, miR-96-5p, miR-323a-3p, miR-203a-3p, miR-32-3p, miR-942-3p, miR-7-5p, and miR-16-2-3p. The detailed information about DEMs is shown in Supplemental Table S2. A heat map and a volcano plot were plotted to display this information (Figures 4(a) and 4(b)).

Moreover, a total of 1543 genes targeted by these candidate DEMs were predicted by the miRNet database. For better visualization, DEMs with their target genes are displayed in a DEM-target gene network in Figure 4(c). In addition, the number of target genes for each DEM is listed in Table 2, and all predicted target genes are listed in Supplemental Table S3.

### 3.5. Prediction of Upstream Transcription Factors of DEMs

In the present study, the upstream transcription factors of candidate DEMs were predicted using FunRich software. Eventually, eight upstream transcription factors were considered statistically significant, including SP1, SP4, YY1, EGR1, E2F1, MEF2A, NFYA, and MYC. This result is presented in Figure 4(d).

### 3.6. Integrated Analysis Results

**Figure 5:**
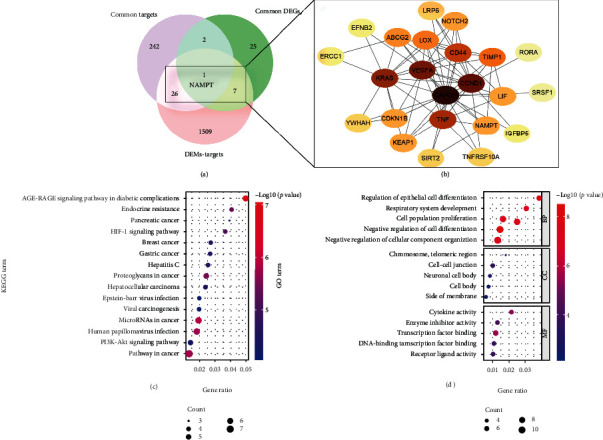
Integrated analysis results. (a) Venn diagram of intersection between the targets of DEMs, common gene targets, and common DEGs. A gene (NAMPT) was found to be shared by multi-datasets. (b) PPI network diagram of 34 genes with a combined score > 0.4. (c) KEGG pathway analysis results of the 34 genes. (d) GO enrichment analysis results of the 34 genes.

In order to find the hub genes, a Venn diagram of intersection between the targets of DEMs, 271 common genes, and 35 common DEGs was constructed (Figure 5(a)). It is noteworthy that a gene (NAMPT) was found to be shared by multisets, implicating a significant role for NAMPT in both osteoporosis and T2DM. As shown in Figure 5(a), 27 intersection genes between targets of DEMs and 271 common genes, and eight intersection genes between targets of DEMs and 35 common DEGs were obtained. The detailed information about these 34 overlapping genes is listed in Table 3, and the PPI network of these genes with a combined score > 0.4 was obtained by Cytoscape (Figure 5(b)). Simultaneously, GO enrichment analysis and KEGG pathway enrichment analysis were also performed. KEGG pathway analysis showed that they were significantly enriched in the AGE-RAGE signaling pathway in diabetic complications, endocrine resistance, the HIF-1 signaling pathway, and the PI3K-Akt signaling pathway (Figure 5(c)). GO analysis showed that they were particularly enriched in negative regulation of cell differentiation, negative regulation of cellular component organization, cell-cell junction, and transcription factor binding (Figure 5(d)). Again, these results highlight the importance of the PI3K-Akt signaling pathway and the AGE-RAGE signaling pathway in diabetic complications.

### 3.7. Construction of miRNA-Gene Network

**Figure 6:**
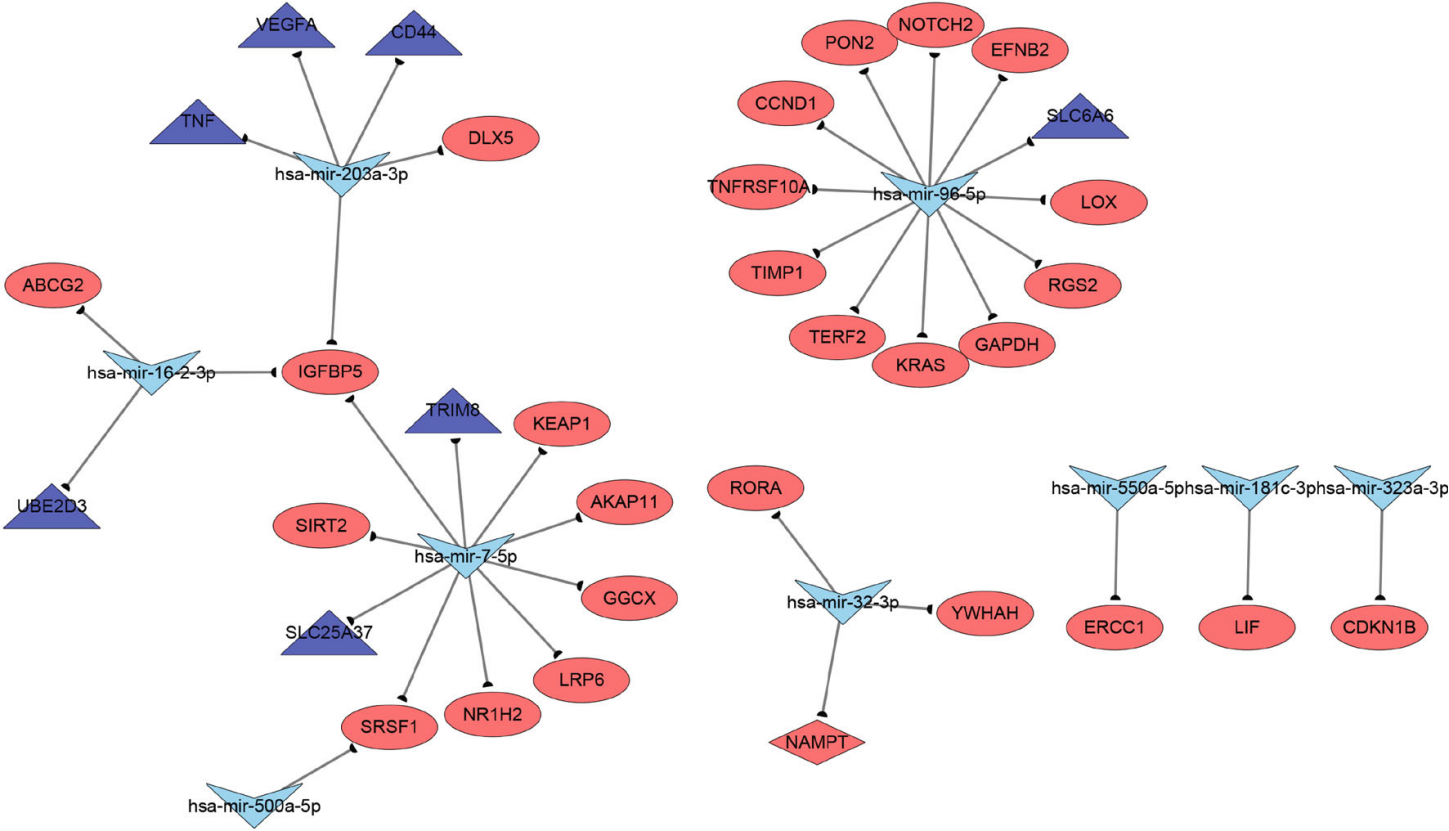
The miRNA-gene regulatory network. miRNAs are represented by a V shape, the common genes are represented by an ellipse shape, the DEGs are represented by triangles, and the gene (NAMPT) is represented by the diamond shape.

To better investigate the molecular mechanisms of these DEMs in T2DM with osteoporotic fracture, the miRNA-gene network was constructed by Cytoscape software and a total of nine miRNAs and 34 genes were obtained (Figure 6). The miR-96-5p and miR-7-5p had the highest degree in DEMs, while the IGFBP5 had the highest degree in hub genes, suggesting which might play crucial roles in the development of T2DM with osteoporotic fracture.

### 3.8. ROC Curves of miRNAs

**Figure 7:**
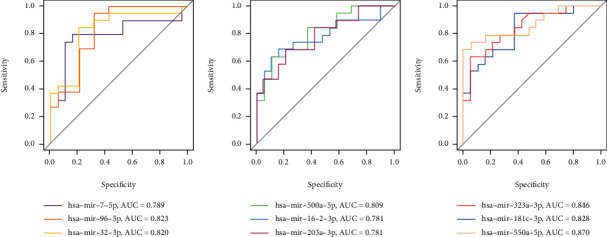
ROC of nine DEMs between T2DM with osteoporotic fracture patients and T2DM patients.

To investigate the efficacy of DEMs in the miRNA-gene network as potential biomarkers of osteoporotic fractures with T2DM, we performed ROC curve analysis of these miRNAs. The expression levels of the DEMs were significantly different between experimental and control individuals (Figure 7). AUC values were used to evaluate the potential of the DEMs as diagnostic markers. The AUC value of miR-550a-5p was 0.870, and it also had the highest accuracy. Moreover, all nine miRNAs had high specificity with AUCs > 0.7, among them six miRNAs had high specificity with AUCs > 0.8. These results indicated that these miRNAs, especially miR-550a-5p, have potential for clinical application.

## 4. Discussion

Available evidence suggests that osteoporosis and T2DM, two common chronic diseases, may coexist and progressively increase in prevalence and are boosted by aging [26]. The risk of fracture of patients with T2DM is increased with longer duration of disease, but the mechanisms remain relatively undefined [5]. Recently, it is easier to reveal the potential disease pathobiology with the development of bioinformatic technology. However, it seems that few studies have explored the molecular mechanisms of bone fragility in T2DM at the genetic level. Therefore, we tried to explore the underlying mechanisms of bone fragility in T2DM using bioinformatic technology to provide some clues for developing dual-purpose prevention methods.

In this study, data from the three public databases were extracted to identify the common gene targets between osteoporosis and T2DM. A total of 271 common gene targets were identified to be further analyzed. The result of GO functional analysis showed that the common genes were closely associated with the blood vessel development, response to growth factor, and cellular response to lipid. A previous study reported that chronic hyperglycemia, the main features of diabetic, caused severe impairment in lipid metabolism [27]. Disruption in lipid metabolism leads to increased level of very low-density lipoprotein (VLDL) and total cholesterol (TC) [28]. VLDL and TC accumulate in subendothelial and endothelial cell layers, which will result in atherosclerosis and narrowing of vascular lumen. A changed vascular supply to the skeleton, in particular cortical bone, could compromise bone formation [29]. Thus, we speculated that the dysregulation of blood vessel development and cellular response to lipid stimulus functions in the process of diabetes led to bone fragility.

The results of KEGG pathway enrichment analysis showed that the common genes were mainly enriched in cytokine-cytokine receptor interaction, T cell differentiation-related pathways, the PI3K-Akt signaling pathway, and the AGE-RAGE signaling pathway in diabetic complications. Emerging evidence has showed that numerous cytokines, including TGF*β*, IL-6, IL-1*β*, and IL-21, are associated with bone remodeling in diabetes [30, 31]. High glucose increases expression of inflammatory factors, and a hyperosmotic environment leads to the overexpression of TLR-4, which can facilitate inflammatory response and affecting osteoblast mineralization [32]. In the previous studies, the activation of the PI3K-Akt signaling pathway has been amply documented to have an association with the proliferation of osteoblasts. A study performed by Ma et al. [33] showed that IRS-1, an activator of PI3K, was capable of enhancing the proliferation of the primary rat osteoblasts. In terms of T2DM, it is clear that the PI3K-Akt signaling pathway is closely related to the pathogenesis of insulin resistance [34]. High-glucose-induced insulin signaling blockade can be attenuated by preventing the inaction of the PI3K-Akt signaling pathway [35]. Hyperglycemia can promote the production of reactive oxygen species by stimulating the PI3K-Akt signaling pathway, and the latter inhibits osteoblast proliferation and differentiation, leading to the development of osteoporosis in T2DM [36]. Moreover, the AGE-RAGE signaling pathway in diabetic complications may also be critical. Hyperglycemic condition leads to excessive accumulation of AGEs, which influence the formation of collagens and ROS, inducing structural changes in bone and impairing bone strength [37]. Higher levels of AGEs have been identified as a biomarker for the increased risk of fractures [38]. Suzuki et al. [39] found that AGEs accumulate in osteoblasts with age and induce apoptosis via ER stress by activating glucose-regulated protein, inositol-requiring protein-1*α* (IRE1*α*), C-Jun n-terminal kinase, etc. These findings have demonstrated that these pathways were associated with bone metabolism in T2DM, which can provide potential directions for the study of the molecular mechanism of T2DM complicated by osteoporosis.

Next, 35 common DEGs between T2DM and osteoporosis were identified by analyzing the GSE35958and GSE43950 datasets. Enrichment analysis results of these DEGs highlighted the role of immune and inflammatory response, which were broadly consistent with the above results. It is noteworthy that the AGE-RAGE signaling pathway in diabetic complications was enriched again, suggesting its importance in diabetic osteoporosis. What is more, a core gene (NAMPT) was found to be shared by multi-datasets. In T2DM, the NAMPT gene codes the protein visfatin, which is critical for beta cell function via mediation of nicotinamide adenine dinucleotide biosynthesis [40]. The single-nucleotide polymorphisms in NAMPT gene were associated with glycemic and metabolic traits as well as T2DM susceptibility [41]. In bone metabolism, it has been reported that NAMPT plays a critical role in osteoblast differentiation through epigenetic augmentation of Runx2 transcription [42] and acts as a negative regulator of RANKL-mediated differentiation of bone marrow macrophages into osteoclasts [43]. However, few studies directly analyze the role of NAMPT in T2DM bone metabolism, which emphasizes its importance in future research.

Then, the analysis of the GSE70318 dataset showed that the expression of various miRNAs has experienced some extent of alteration in T2DM patients with osteoporotic fracture compared to T2DM patients. As reported in recent studies, the expression of miRNA can be modulated by transcription factors [44, 45], and thus, we filtered out possible transcription factors. Specificity protein 1 (SP1), a C_2_H_2_-type zinc-finger transcription factor [46], is the most common transcription factor. A previous study has shown that a polymorphism that affects an SP1 binding site in the COLIA1 gene is associated with reduced bone mineral density and an increased risk of osteoporotic fracture in postmenopausal population [47]. Following that, Yu et al. [48] suggested that SP1 played a role in human osteoblast differentiation and mineralization by SP1-dependent transactivation of FZD1. In addition, experiment has also shown that insulin- and glucose-responsive genes can be regulated by a dynamic interplay between glycosylation and phosphorylation of SP1 [49]. These evidences indicate that transcription factor SP1 may contribute to the etiopathogenesis of bone fragility in T2DM. As for other remaining transcription factors, there is no clear relationship between them and bone metabolism in T2DM, but it is worthy of further exploration.

Moreover, by constructing DEM-gene network, we found that most of hub genes could be potentially targeted by miR-96-5p and miR-7-5p. Prior research demonstrated that miR-96-5p indirectly regulated glutathione levels, a key antioxidant responsible for eliminating the damaging oxidative stress-related reactive oxygen species [50]. However, Yu et al. [51] observed that miR-96-5p levels were markedly low under high-glucose conditions. The serum levels of miR-96-5p in T2DM with osteoporotic fracture are almost 3 times lower than control T2DM subjects, which may hint that diabetes-induced oxidative stress levels regulated by miR-96-5p might be a potential path mechanism for the higher fracture rate in T2DM individuals [52]. Despite the miR-96-5p, miR-7-5p has been reported to have the ability of inducting osteoblast differentiation and upregulating the expression of osteogenic differentiation-related proteins, including Runx2, ALP, collagen type I alpha 1 (Col1a1), and OCN [53]. The downregulation of miR-7-5p may result in decreased bone mass and osteoblastogenesis, but its role in diabetic osteoporosis needs further studies.

Proper recognition of populations at increased fracture risk is indispensable, but the risk of fracture in T2DM individuals tends to be underestimated. At present, researchers generally believe that the pathophysiology of osteoporosis ultimately causing fractures had a heterogeneous etiology with different miRNA expression patterns. Therefore, we performed ROC curve analysis of nine DEMs in the DEM-gene network. Results show that the miR-550a-5p has the highest score of AUC, and it has been verified to inhibit osteogenic differentiation in vitro [25]. Additionally, miR-181c has been proved to engage the progression of bone loss in osteoporosis and the bone homeostasis mediated by osteoclasts and osteoblasts [54]. Other remaining miRNAs (like miR-7-5p and miR-96-5p) have also been shown to be related to bone metabolism and believe that they are worthy of further exploration.

IGFBP5 (insulin-like growth factor binding protein 5) is selected as the key genes in the miRNA-gene network. Insulin-like growth factors (IGFs) have regulatory effects in bone cells, such as regulating cell proliferation, differentiation, and apoptosis, and are controlled by their cognate receptors, IGF-binding proteins (IGFBPs), and IGFBP proteases [55]. It has been shown that IGFBP5 controls osteoblast differentiation and osteoblast-osteoclast cross-talk [56]. In support of this, IGFBP5 treatment increased bone formation parameters in vitro and in vivo in osteoblasts derived from IGF-I knockout mice [57]. At the same time, IGFBP5 can enhance insulin sensitivity to exert antidiabetic effects by inhibiting the expression of the thioredoxin-interacting protein (TXNIP) and arrestin domain-containing 4 (ARRDC4) [58]. Therefore, the expression of IGFBP5 may simultaneously regulate blood glucose metabolism and bone metabolism, engaging in bone fragility in T2DM.

To our knowledge, few studies have explored the common molecular mechanism between osteoporosis and T2DM by advanced bioinformatics methods. Due to the high rate of osteoporotic fracture in T2DM, we explored the pathological mechanisms of bone fragility in T2DM based on the joint analysis of multi-databases and multi-datasets for the first time, which helped to develop dual-purpose prevention methods. However, there are several limitations to our present study. First of all, the study was performed based only on the serum samples not bone tissue, so studies aimed at bone tissue in T2DM are needed. In addition, the miRNA-gene interactions were only based on predictions from public databases, and further studies with cellular and animal experimental validations are still needed to validate our analysis.

## 5. Conclusion

In this study, we explored the common pathomechanism between T2DM and osteoporosis and found that the PI3K/AKT signaling pathway and the AGE-RAGE signaling pathway in diabetic complications might play crucial roles in the common pathomechanism. Simultaneously, a miRNA-gene network was constructed and identified some potential miRNAs and genes, which may participate in the pathogenesis of bone fragility in T2DM. We hope that these findings can contribute to deepen our knowledge on the pathologic mechanism of bone fragility in T2DM.

## Figures and Tables

**Table 1:** The detailed information about 35 common DEGs in the GSE35958 and GSE43950.

Gene/dataset	GSE35958		GSE43950	
	Adj. P value	logFC	Adj. P value	logFC
TIMP2	0.0301804	1.067603	0.000286	2.18
ALDH3B1	0.0412961	1.195918	0.044747	1.82
EXOC7	0.0235592	1.249935	0.021148	1.04
ICAM1	0.01655111	1.812343	0.046365	1.31
WIPF1	0.0225064	1.320364	0.002505	1.04
SVIL	0.0456309	1.407409	0.028703	1.14
PLAUR	0.0184105	1.46844	0.007675	3.34
SLC25A37	0.0315111	1.485996	0.040526	2.76
LAPTM5	0.0261593	1.495128	0.047332	1.81
MAPKAP1	0.0270001	1.511965	0.02719	2.12
ZDHHC5	0.0212057	1.540676	0.038415	1.32
MIA3	0.0086971	1.609845	0.038795	1.59
CPD	0.0355536	1.635179	0.033381	2.11
NAMPT	0.0208943	1.639585	0.015879	3.95
IL1R1	0.0196939	1.678116	0.021148	1.42
BCL6	0.0263706	1.685134	0.013304	2.34
CD44	0.0061623	1.697591	0.047805	1.11
OGDH	0.0234526	1.814095	0.008177	1.4
TNF	0.0336169	1.917344	0.025653	3.47
SAR1B	0.0126974	2.026727	0.032663	1.08
MAP3K2	0.0023339	2.344297	0.031489	1.77
PDE4B	0.0277296	2.401498	0.042734	2.7
UBE2D3	0.0439363	2.816778	0.03572	2.26
SBF2	0.0147589	2.944771	0.039608	2.46
TRIM8	0.0018391	2.956019	0.039673	1.13
BHLHE40	0.0005991	3.244912	0.021148	3.42
VEGFA	0.012642	3.259643	0.009092	2.91
SLC6A6	0.0252533	3.431333	0.013647	1.31
TRIB1	0.0221217	3.47739	0.013547	2.79
RAB7A	0.0489256	3.486461	0.017748	1.25
BEST1	0.0364247	3.592602	0.006973	2.54
CSGALNACT2	0.0080592	3.73142	0.038795	2.19
RARA	0.000817	3.98471	0.044649	1.53
EIF5B	0.0039468	-2.89269	0.01708	-1.07
DAZAP1	0.0340059	-1.07479	0.039575	-1.04

**Table 2:** Potential target genes of the nine DEMs.

miRNAs	Number of targets
hsa-miR-96-5p	201
hsa-miR-7-5p	578
hsa-miR-203a-3p	308
hsa-miR-323a-3p	75
hsa-miR-32-3p	124
hsa-miR-16-2-3p	85
hsa-miR-181c-3p	32
hsa-miR-500a-5p	145
hsa-miR-550a-5p	95
hsa-miR-942-3p	69
Total	1543

**Table 3:** The detailed information about the 34 hub genes and their regulated DEMs.

Gene	Full name	DEMs
CCND1	G1/S-specific cyclin-D1	hsa-miR-96-5p
EFNB2	Ephrin-B2	hsa-miR-96-5p
GAPDH	Glyceraldehyde-3-phosphate dehydrogenase	hsa-miR-96-5p
KRAS	GTPase Kras	hsa-miR-96-5p
LOX	Protein-lysine 6-oxidase	hsa-miR-96-5p
NOTCH2	Neurogenic locus notch homolog protein 2	hsa-miR-96-5p
PON2	Serum paraoxonase/arylesterase 2	hsa-miR-96-5p
RGS2	Regulator of G-protein signaling 2	hsa-miR-96-5p
TERF2	Telomeric repeat-binding factor 2	hsa-miR-96-5p
TIMP1	Metalloproteinase inhibitor 1	hsa-miR-96-5p
TNFRSF10A	Tumor necrosis factor receptor superfamily member 10A	hsa-miR-96-5p
GGCX	Vitamin K-dependent gamma-carboxylase	hsa-miR-7-5p
IGFBP5	Insulin-like growth factor-binding protein 5	hsa-miR-7-5p
		hsa-miR-203a-3p
		hsa-miR-16-2-3p
LRP6	Low-density lipoprotein receptor-related protein 6	hsa-miR-7-5p
SRSF1	Serine/arginine-rich splicing factor 1	hsa-miR-7-5p
		hsa-miR-500a-5p
NR1H2	Oxysterols receptor LXR-beta	hsa-miR-7-5p
KEAP1	Kelch-like ECH-associated protein 1	hsa-miR-7-5p
AKAP11	A-kinase anchor protein 11	hsa-miR-7-5p
SIRT2	NAD-dependent protein deacetylase sirtuin-2	hsa-miR-7-5p
DLX5	Homeobox protein DLX-5	hsa-miR-203a-3p
CDKN1B	Cyclin-dependent kinase inhibitor 1B	hsa-miR-323a-3p
RORA	RAR-related orphan receptor alpha	hsa-miR-32-3p
YWHAH	Tryptophan 5-monooxygenase activation protein eta	hsa-miR-32-3p
NAMPT	Nicotinamide phosphoribosyltransferase	hsa-miR-32-3p
ABCG2	ATP-binding cassette sub-family G member 2	hsa-miR-16-2-3p
LIF	Lif, interleukin 6 family cytokine	hsa-miR-181c-3p
ERCC1	DNA excision repair protein ERCC-1	hsa-miR-550a-5p
SLC6A6	Solute carrier family 6 member 6	hsa-miR-96-5p
SLC25A37	Solute carrier family 25 member 37	hsa-miR-7-5p
TRIM8	Tripartite motif containing 8	hsa-miR-7-5p
TNF	Tumor necrosis factor	hsa-miR-203a-3p
VEGFA	Vascular endothelial growth factor A	hsa-miR-203a-3p
NAMPT	Nicotinamide phosphoribosyltransferase	hsa-miR-32-3p
UBE2D3	Ubiquitin conjugating enzyme E2 D3	hsa-miR-16-2-3p
CD44	CD44 antigen	hsa-miR-203a-3p

## Data Availability

No data were used to support this study.

## Supplementary Materials

Supplementary Materials Supplemental Table S1: the 271 common genes between T2DM and osteoporosis gained from the three public databases. Supplemental Table S2: DEMs between T2DM with osteoporotic fracture and T2DM plasma from the GSE70318 dataset. Supplemental Table S3: target genes of DEMs predicted by miRNet.
