# Supplementary material for: Integrated Analysis of Crucial Genes and miRNAs Associated with Osteoporotic Fracture of Type 2 Diabetes
Source: Biomed Res Int. 2022 Aug 10;2022:3921570. doi: 10.1155/2022/3921570 (PMC9385370; doi:10.1155/2022/3921570)
Supplement: Supplementary Materials — Supplemental Table S1: the 271 common genes between T2DM and osteoporosis gained from the three public databases. Supplemental Table S2: DEMs between T2DM with osteoporotic fracture and T2DM plasma from the GSE70318 dataset. Supplemental Table S3: target genes of DEMs predicted by miRNet. [file 3921570.f1.pdf]

Table S1 The 271 common genes between T2DM and osteoporosis gained from public databases.

No.	Gene	No.	Gene	No.	Gene	No.	Gene
1	TGFB1	69	TTR	137	RETN	205	HOXC8
2	COL1A1	70	ALDH2	138	TWIST1	206	GPATCH1
3	CYP24A1	71	INHA	139	PON2	207	PROKR2
4	ENO1	72	NAMPT	140	SMPD3	208	TNFRSF13B
5	GAPDH	73	EPO	141	MSX2	209	STARD3NL
6	PLEK	74	KRT14	142	SERPINF1	210	TERF2
7	VCL	75	SLC10A2	143	FGF4	211	IAPP
8	MMP2	76	RORA	144	KRAS	212	GTPBP1
9	HMOX1	77	HNF1A	145	IBSP	213	PLVAP
10	MAPK1	78	MAPK13	146	BHLHE40	214	COMP
11	NFKB1	79	SULT1E1	147	THPO	215	SMPD2
12	NFKBIA	80	PNPLA3	148	CX3CL1	216	TPH1
13	CCND1	81	SRSF1	149	KITLG	217	F9
14	IL4	82	TG	150	SIRT2	218	LYVE1
15	CCL2	83	FGF23	151	GCK	219	GNRHR
16	IFNG	84	FCGRT	152	MYOD1	220	MAPK12
17	MPO	85	LMNB1	153	SCT	221	FBL
18	GSR	86	HDAC7	154	ADH1A	222	GHSR
19	CDKN1B	87	EFNB2	155	STS	223	ATP12A
20	VWF	88	CYP27A1	156	ENPP2	224	HS6ST1
21	ABCG2	89	NOTCH2	157	TSHB	225	BNIPL
22	NR3C1	90	AKAP11	158	SLC6A4	226	MC3R
23	CTSD	91	NTS	159	SCTR	227	XYLT2
24	IL2	92	CD79A	160	RAMP2	228	PALB2
25	CRP	93	XYLT1	161	BMP15	229	AAAS
26	SPARC	94	CYP26A1	162	CDK4	230	PRDM2
27	RARA	95	ALOX12	163	MGP	231	TCN2
28	PLAT	96	TBX21	164	FST	232	CHRD
29	THBS1	97	CHGA	165	APOE	233	CEACAM1
30	GCLC	98	LGALS3	166	HSPB1	234	PPP3R1
31	TIMP1	99	SOX4	167	MIF	235	SLCO1B1
32	TNFSF10	100	NOD1	168	TNFRSF1A	236	KCNQ1
33	C3	101	PRTN3	169	RGS2	237	AFM
34	FABP4	102	NR1H2	170	SOX9	238	GNA11
35	BMP4	103	HDAC5	171	CFTR	239	CETP
36	CCNB1	104	SORT1	172	INHBA	240	AEBP1
37	IGFBP5	105	SLC11A1	173	PPP1R15A	241	CTSG
38	HGF	106	RAC2	174	PTGIS	242	DLL4
39	SIRT1	107	ABCC6	175	SELL	243	APEX1
40	IL5	108	MYH9	176	DUSP1	244	LRP6
41	MMP7	109	TGM1	177	TRAF2	245	RDH10

42	PER2	110	DLX5	178	APOB	246	TRAF5
43	CYP2C9	111	PTPN12	179	TGFB3	247	MTNR1B
44	SERPINE1	112	UBE3A	180	MMP13	248	FAAH
45	POU5F1	113	CD4	181	NR0B2	249	SETDB1
46	MAPK14	114	APOC3	182	GRN	250	KDM6B
47	EIF2S1	115	HTRA2	183	TLR2	251	TNNT2
48	MDM2	116	PEPD	184	IL1RN	252	CD82
49	APOA1	117	HBA2	185	DCN	253	OSM
50	SULT2A1	118	MEFV	186	PON1	254	ERCC1
51	ITGAV	119	ACVR2A	187	XBP1	255	HOXC6
52	IRF1	120	M6PR	188	NGFR	256	YWHAH
53	GZMB	121	MSTN	189	KLRK1	257	MMP8
54	OXT	122	GDF11	190	TNFRSF10A	258	OTC
55	CD68	123	AMH	191	GDF15	259	CSK
56	GOT2	124	CMA1	192	EPOR	260	ARF5
57	VTN	125	DVL2	193	CD38	261	INPP4A
58	LIF	126	GGCX	194	TGFBR3	262	KAT2A
59	MYOG	127	TBL1X	195	SLC40A1	263	WNK4
60	NT5E	128	FLT3	196	IGFBP2	264	WNT3
61	FOXA1	129	SKP1	197	PDGFRA	265	STUB1
62	GLI1	130	NOX3	198	CHI3L1	266	CDC42SE1
63	JAG1	131	RBX1	199	IRAK2	267	CEACAM5
64	SLC10A1	132	ATP7B	200	ACLY	268	MYF6
65	KEAP1	133	IRAK3	201	LRP1	269	ALDH7A1
66	CYP27B1	134	WNT16	202	CTSC	270	PKD2
67	CSF3	135	APC	203	LOX	271	KDM4B
68	NR1D1	136	TTPA	204	SFRP1		

Table S2 DEMs between T2DM with osteoporotic fracture and T2DM plasma from GSE70318 dataset.

ID	adj.P.Val	P.Value	t	B	logFC
hsa-miR-550a-5p	8.98E-05	5.87E-07	5.891	6.049	-4.4858
hsa-miR-500a-5p	0.021558	0.000594	3.7176	-0.465	-1.8905
hsa-miR-181c-3p	0.021558	0.000667	3.678	-0.574	-1.4647
hsa-miR-96-5p	0.021558	0.00069	3.6666	-0.605	-1.3447
hsa-miR-323a-3p	0.021558	0.000705	3.6597	-0.624	-1.2858
hsa-miR-203a	0.033925	0.00155	3.3873	-1.353	-3.1995
hsa-miR-32-3p	0.033925	0.00146	3.4093	-1.296	-1.1111
hsa-miR-942	0.034032	0.00178	3.3392	-1.479	-2.1621
hsa-miR-7-5p	0.039513	0.00258	3.2065	-1.82	-1.3958
hsa-miR-16-2-3p	0.039513	0.00241	3.231	-1.758	-1.0295
hsa-let-7g-5p	0.067925	0.00533	2.9406	-2.479	-0.9868
hsa-let-7i-5p	0.067925	0.00528	2.9437	-2.471	-0.9058
hsa-miR-19b-1-5p	0.073966	0.0079	2.7908	-2.833	-2.1637
hsa-miR-143-5p	0.073966	0.0071	2.8317	-2.738	-1.3016
hsa-miR-181c-5p	0.073966	0.00822	2.7756	-2.869	-1.2674
hsa-miR-141-3p	0.073966	0.00658	2.8608	-2.669	-1.2668
hsa-miR-486-5p	0.073966	0.00786	2.7925	-2.829	-1.0432
hsa-miR-382-3p	0.074305	0.012	-2.6276	-3.205	2.8032
hsa-miR-301a-3p	0.074305	0.0108	2.6676	-3.116	-1.1495
hsa-miR-21-3p	0.074305	0.00962	2.7142	-3.01	-1.0889
hsa-miR-106b-5p	0.074305	0.0121	2.6225	-3.217	-1.0068
hsa-miR-191-5p	0.074305	0.0109	2.6647	-3.122	-0.9858
hsa-let-7b-5p	0.074305	0.0111	2.6579	-3.138	-0.9721
hsa-miR-532-3p	0.074305	0.0107	2.6708	-3.109	-0.8695
hsa-miR-92a-3p	0.074305	0.0114	2.6475	-3.161	-0.8147
hsa-miR-941	0.077697	0.0132	2.5889	-3.291	-1.5911
hsa-miR-486-3p	0.078274	0.0153	2.5282	-3.424	-1.1642
hsa-miR-106a-5p	0.078274	0.0138	2.5697	-3.334	-0.9563
hsa-miR-629-5p	0.078274	0.0147	2.5463	-3.385	-0.9547
hsa-miR-181a-5p	0.078274	0.015	2.5376	-3.404	-0.7358
hsa-miR-375	0.080261	0.0163	2.5046	-3.475	-1.3042
hsa-miR-378a-5p	0.082401	0.0172	2.4808	-3.526	-1.0753
hsa-miR-550a-3p	0.08293	0.0193	2.4332	-3.628	-0.9842
hsa-miR-20a-5p	0.08293	0.0185	2.4519	-3.588	-0.9226
hsa-miR-146b-5p	0.08293	0.019	2.4409	-3.612	-0.8953
hsa-miR-16-5p	0.08293	0.0195	2.4295	-3.635	-0.8868
hsa-miR-651	0.083467	0.0202	2.4155	-3.665	-1.0242
hsa-miR-545-3p	0.084241	0.0257	2.3133	-3.876	-1.3832
hsa-miR-199b-5p	0.084241	0.0295	2.2537	-3.996	-1.3474
hsa-miR-301b	0.084241	0.0209	2.4005	-3.696	-1.2474
hsa-miR-324-5p	0.084241	0.0318	2.221	-4.061	-1.1489

hsa-miR-454-3p	0.084241	0.0257	2.313	-3.877	-0.9895
hsa-miR-627	0.084241	0.0319	2.2198	-4.063	-0.9679
hsa-miR-144-3p	0.084241	0.0229	2.362	-3.776	-0.9368
hsa-miR-93-5p	0.084241	0.0226	2.3674	-3.765	-0.9342
hsa-miR-18b-5p	0.084241	0.0301	2.245	-4.013	-0.92
hsa-miR-451a	0.084241	0.026	2.3094	-3.884	-0.9058
hsa-miR-18a-5p	0.084241	0.0303	2.2421	-4.019	-0.8842
hsa-miR-98-5p	0.084241	0.0219	2.3808	-3.737	-0.8532
hsa-miR-374a-5p	0.084241	0.0263	2.3045	-3.894	-0.8379
hsa-miR-363-3p	0.084241	0.0247	2.3301	-3.842	-0.8268
hsa-miR-25-3p	0.084241	0.0271	2.2903	-3.923	-0.8195
hsa-miR-140-3p	0.084241	0.0309	2.2345	-4.034	-0.7826
hsa-miR-19b-3p	0.084241	0.0308	2.2351	-4.033	-0.7642
hsa-miR-15b-3p	0.084241	0.0279	2.2787	-3.946	-0.7363
hsa-miR-30e-5p	0.084241	0.0281	2.2754	-3.953	-0.7205
hsa-miR-15a-5p	0.084241	0.0267	2.2966	-3.91	-0.6921
hsa-miR-660-5p	0.084241	0.0294	2.2552	-3.993	-0.6516
hsa-miR-17-5p	0.085766	0.0331	2.2045	-4.093	-0.9795
hsa-miR-185-5p	0.085982	0.0337	2.196	-4.11	-0.8053
hsa-miR-132-3p	0.088758	0.036	2.1675	-4.165	-0.84
hsa-miR-144-5p	0.088758	0.0358	2.1695	-4.161	-0.6632
hsa-miR-26b-5p	0.095601	0.04	2.1202	-4.256	-0.7895
hsa-miR-29b-3p	0.095601	0.04	2.1203	-4.256	-0.7389
hsa-miR-19a-3p	0.096798	0.0411	2.1077	-4.28	-0.7084
hsa-miR-93-3p	0.097191	0.0419	2.0989	-4.296	-0.8632
hsa-miR-20b-5p	0.098009	0.0436	2.0817	-4.328	-1.0484
hsa-miR-101-3p	0.098009	0.043	2.0876	-4.317	-0.6879
hsa-miR-324-3p	0.100267	0.0459	2.0581	-4.372	-0.6984
hsa-miR-425-5p	0.100267	0.0458	2.0589	-4.371	-0.6853
hsa-miR-186-5p	0.100363	0.0466	2.0512	-4.385	-0.6863
hsa-miR-369-3p	0.100743	0.0474	-2.0431	-4.4	1.2205
hsa-miR-21-5p	0.10304	0.0492	2.0264	-4.431	-0.5805
hsa-miR-320b	0.104482	0.0505	2.0137	-4.454	-0.5989
hsa-miR-423-5p	0.105143	0.0515	2.0046	-4.47	-0.5758
hsa-miR-342-3p	0.106768	0.053	1.9913	-4.494	-0.59
hsa-miR-210	0.111376	0.0561	1.9655	-4.541	-0.7784
hsa-miR-425-3p	0.11274	0.0582	1.9477	-4.572	-0.6916
hsa-miR-145-5p	0.11274	0.0576	1.9525	-4.564	-0.5637
hsa-miR-196b-5p	0.118286	0.0641	1.9021	-4.652	-1.0484
hsa-miR-590-3p	0.118286	0.0661	1.8876	-4.677	-0.9174
hsa-miR-143-3p	0.118286	0.0665	1.8845	-4.683	-0.8658
hsa-miR-181b-5p	0.118286	0.0664	1.8852	-4.682	-0.8326
hsa-miR-18a-3p	0.118286	0.0662	1.8868	-4.679	-0.7721
hsa-miR-194-5p	0.118286	0.0646	1.898	-4.66	-0.6868

hsa-miR-29a-3p	0.118286	0.0665	1.8845	-4.683	-0.6095
hsa-miR-200b-3p	0.124522	0.0708	1.8542	-4.735	-0.9305
hsa-miR-24-3p	0.125788	0.0723	1.8437	-4.752	-0.6284
hsa-miR-1908*	0.129052	0.0751	-1.8257	-4.783	1.3379
hsa-miR-376c-3p	0.133714	0.0801	-1.7941	-4.835	0.9842
hsa-miR-484	0.133714	0.0799	1.795	-4.834	-0.5868
hsa-miR-874*	0.133714	0.0804	1.792	-4.839	-0.5695
hsa-miR-532-5p	0.137342	0.0835	1.7734	-4.87	-0.7395
hsa-miR-100-5p	0.141464	0.0883	1.7456	-4.915	-0.69
hsa-miR-145-3p	0.141464	0.0888	1.7427	-4.919	-0.6421
hsa-miR-32-5p	0.141464	0.0879	1.7475	-4.912	-0.6132
hsa-miR-22-3p	0.144019	0.0917	1.7262	-4.946	-0.5047
hsa-miR-128*	0.144019	0.0922	1.7233	-4.95	-0.4842
hsa-miR-598*	0.14928	0.0985	1.6898	-5.004	-0.8689
hsa-miR-361-3p	0.14928	0.0975	1.695	-4.995	-0.6332
hsa-let-7d-3p	0.14928	0.0984	1.6906	-5.002	-0.4505
hsa-miR-652-3p	0.15456	0.103	1.6669	-5.039	-0.5574
hsa-miR-215*	0.157733	0.106	1.6514	-5.063	-0.6563
hsa-miR-192-5p	0.160707	0.109	1.6367	-5.086	-0.6258
hsa-miR-22-5p	0.162715	0.112	1.6252	-5.103	-0.4789
hsa-miR-320c	0.163374	0.113	1.6181	-5.114	-0.5021
hsa-miR-222-3p	0.166043	0.116	1.6047	-5.134	-0.4989
hsa-miR-320a	0.170088	0.12	1.5871	-5.161	-0.47
hsa-miR-223-5p	0.170597	0.122	1.5806	-5.17	-0.5684
hsa-miR-330-3p	0.174981	0.127	1.5574	-5.204	-1.0526
hsa-let-7i-3p	0.174981	0.127	1.5585	-5.203	-0.6368
hsa-miR-154-5p	0.176087	0.129	-1.5492	-5.216	1.5253
hsa-let-7a-3p	0.181637	0.134	1.5277	-5.247	-0.5126
hsa-let-7e-5p	0.184085	0.138	-1.5109	-5.271	0.5642
hsa-miR-214-3p	0.184085	0.137	1.5155	-5.265	-0.5126
hsa-miR-331-3p	0.185824	0.141	1.5011	-5.285	-0.6026
hsa-miR-493-5p	0.188221	0.144	-1.4893	-5.302	1.6326
hsa-miR-130a-3p	0.19361	0.149	1.4691	-5.33	-0.4442
hsa-miR-205-5p	0.249263	0.194	1.3205	-5.527	-0.5305
hsa-miR-320d	0.271368	0.213	1.2652	-5.595	-0.4132
hsa-miR-190a	0.310413	0.245	1.178	-5.697	-0.6679
hsa-miR-624-5p	0.336064	0.268	1.1228	-5.759	-0.6205
hsa-miR-382-5p	0.338369	0.272	-1.1132	-5.769	0.4747
hsa-miR-30e-3p	0.361457	0.293	1.0652	-5.82	-0.4068
hsa-miR-582-3p	0.362709	0.296	1.0576	-5.827	-0.6805
hsa-miR-181d	0.368808	0.304	1.0405	-5.845	-0.9132
hsa-miR-27a-3p	0.368808	0.306	1.0361	-5.849	-0.3632
hsa-miR-502-5p	0.373394	0.312	1.0226	-5.863	-0.5868
hsa-miR-376a-3p	0.38337	0.33	-0.9851	-5.899	0.5905

hsa-miR-136-5p	0.38337	0.329	-0.9872	-5.897	0.48
hsa-miR-136-3p	0.38337	0.331	0.9841	-5.9	-0.4721
hsa-miR-339-5p	0.38337	0.324	-0.9981	-5.887	0.4184
hsa-let-7b-3p	0.414994	0.361	0.9241	-5.956	-0.2789
hsa-miR-34a-5p	0.426929	0.374	0.8988	-5.978	-0.4958
hsa-miR-127-3p	0.455137	0.402	-0.8474	-6.022	0.9537
hsa-miR-487b	0.456391	0.406	-0.84	-6.028	0.45
hsa-miR-133b	0.460719	0.416	-0.8224	-6.043	0.3437
hsa-miR-155-5p	0.460719	0.415	0.8233	-6.042	-0.2853
hsa-miR-410	0.466849	0.424	-0.8072	-6.055	0.3395
hsa-miR-95	0.515184	0.471	0.7268	-6.115	-0.2516
hsa-miR-181a-3p	0.587651	0.542	-0.6155	-6.189	0.4095
hsa-miR-576-3p	0.647252	0.601	0.5274	-6.238	-0.2163
hsa-miR-1227-3p	0.678822	0.634	0.479	-6.262	-0.34
hsa-miR-193b-3p	0.681714	0.642	-0.4689	-6.267	0.2537
hsa-miR-642a-5p	0.704358	0.668	0.4326	-6.283	-0.2711
hsa-miR-589-5p	0.765149	0.73	0.3473	-6.316	-0.2968
hsa-miR-548a-3p	0.798084	0.767	-0.2985	-6.332	0.1811
hsa-miR-188-3p	0.81571	0.789	0.2693	-6.34	-0.1463
hsa-miR-135a-5p	0.834661	0.813	0.2383	-6.348	-0.1058
hsa-miR-342-5p	0.848176	0.832	-0.2141	-6.353	0.0937
hsa-miR-495-3p	0.854626	0.843	-0.1987	-6.357	0.1189
hsa-miR-542-5p	0.932054	0.926	0.0935	-6.372	-0.1037
hsa-miR-377-3p	0.983947	0.984	0.0202	-6.376	-0.0116

Table S3 Target genes of DEMs predicted by miRNet.

ID	Target	ID	Target	ID	Target
hsa-mir-96-5p	ACTN4	hsa-mir-7-5p	UBE2D4	hsa-mir-323a-3p	WDR45B
hsa-mir-96-5p	ACY1	hsa-mir-7-5p	SLC25A39	hsa-mir-323a-3p	CAMK1D
hsa-mir-96-5p	ADCY6	hsa-mir-7-5p	LUC7L2	hsa-mir-323a-3p	NLGN4X
hsa-mir-96-5p	ADSS	hsa-mir-7-5p	ASB2	hsa-mir-323a-3p	RAP2C
hsa-mir-96-5p	ABCD1	hsa-mir-7-5p	C11orf24	hsa-mir-323a-3p	ENPP5
hsa-mir-96-5p	ALK	hsa-mir-7-5p	POLE3	hsa-mir-323a-3p	PHC3
hsa-mir-96-5p	XIAP	hsa-mir-7-5p	DNAJC10	hsa-mir-323a-3p	KCTD10
hsa-mir-96-5p	CCND1	hsa-mir-7-5p	ARMCX6	hsa-mir-323a-3p	ZNF566
hsa-mir-96-5p	BCL2	hsa-mir-7-5p	TRMT13	hsa-mir-323a-3p	LIN52
hsa-mir-96-5p	BDNF	hsa-mir-7-5p	MIER2	hsa-mir-323a-3p	PDZD8
hsa-mir-96-5p	ZFP36L1	hsa-mir-7-5p	NDFIP2	hsa-mir-323a-3p	IFFO2
hsa-mir-96-5p	CASP2	hsa-mir-7-5p	ARL15	hsa-mir-323a-3p	ACVR1C
hsa-mir-96-5p	SERPINH1	hsa-mir-7-5p	MINDY2	hsa-mir-323a-3p	TRUB1
hsa-mir-96-5p	CCND2	hsa-mir-7-5p	TMEM106B	hsa-mir-323a-3p	SESN3
hsa-mir-96-5p	CCNG1	hsa-mir-7-5p	CRLS1	hsa-mir-323a-3p	KIAA2026
hsa-mir-96-5p	SCARB1	hsa-mir-7-5p	TET2	hsa-mir-323a-3p	SDE2
hsa-mir-96-5p	CDKN1A	hsa-mir-7-5p	UBE2R2	hsa-mir-323a-3p	AGO3
hsa-mir-96-5p	CHML	hsa-mir-7-5p	DUSP23	hsa-mir-323a-3p	METTL15
hsa-mir-96-5p	CPE	hsa-mir-7-5p	SLC35A5	hsa-mir-323a-3p	CNEP1R1
hsa-mir-96-5p	CSNK1D	hsa-mir-7-5p	RBM23	hsa-mir-323a-3p	PGM2L1
hsa-mir-96-5p	CTSB	hsa-mir-7-5p	TMEM38B	hsa-mir-32-3p	AZF1
hsa-mir-96-5p	DDIT3	hsa-mir-7-5p	VPS13D	hsa-mir-32-3p	BAAT
hsa-mir-96-5p	DYNC1H1	hsa-mir-7-5p	GOLPH3L	hsa-mir-32-3p	CAPZA2
hsa-mir-96-5p	ECT2	hsa-mir-7-5p	PHF10	hsa-mir-32-3p	CREBL2
hsa-mir-96-5p	EEF1A1	hsa-mir-7-5p	C5orf22	hsa-mir-32-3p	E2F3
hsa-mir-96-5p	EFNB2	hsa-mir-7-5p	LRRC59	hsa-mir-32-3p	FANCF
hsa-mir-96-5p	EIF4EBP2	hsa-mir-7-5p	YOD1	hsa-mir-32-3p	FBLN2
hsa-mir-96-5p	EIF4G1	hsa-mir-7-5p	KIF16B	hsa-mir-32-3p	FOXC1
hsa-mir-96-5p	FOXO1	hsa-mir-7-5p	PACS1	hsa-mir-32-3p	HMGN1
hsa-mir-96-5p	FOXO3	hsa-mir-7-5p	NDC1	hsa-mir-32-3p	HOXA10
hsa-mir-96-5p	FLII	hsa-mir-7-5p	DNAJC11	hsa-mir-32-3p	HOXD11
hsa-mir-96-5p	GABRB1	hsa-mir-7-5p	AGK	hsa-mir-32-3p	HSPA8
hsa-mir-96-5p	GAPDH	hsa-mir-7-5p	RNF114	hsa-mir-32-3p	RBPJ
hsa-mir-96-5p	GDNF	hsa-mir-7-5p	NXT2	hsa-mir-32-3p	IL7R
hsa-mir-96-5p	GSK3B	hsa-mir-7-5p	RCC2	hsa-mir-32-3p	KIF5B
hsa-mir-96-5p	GTF2A1	hsa-mir-7-5p	C20orf24	hsa-mir-32-3p	LYN
hsa-mir-96-5p	HMGCS1	hsa-mir-7-5p	PDGFC	hsa-mir-32-3p	TM4SF1
hsa-mir-96-5p	HNRNPA1	hsa-mir-7-5p	SERTAD4	hsa-mir-32-3p	NHS
hsa-mir-96-5p	HOXA5	hsa-mir-7-5p	MEPCE	hsa-mir-32-3p	PHKA1
hsa-mir-96-5p	HOXA9	hsa-mir-7-5p	BCCIP	hsa-mir-32-3p	TWF1
hsa-mir-96-5p	HTR1B	hsa-mir-7-5p	EIF5A2	hsa-mir-32-3p	RANGAP1
hsa-mir-96-5p	IARS	hsa-mir-7-5p	POLE4	hsa-mir-32-3p	RORA

hsa-mir-96-5p	IGF1R	hsa-mir-7-5p	KCNK13	hsa-mir-32-3p	SMTN
hsa-mir-96-5p	ITPR3	hsa-mir-7-5p	JPH1	hsa-mir-32-3p	SUMO2
hsa-mir-96-5p	KRAS	hsa-mir-7-5p	UBQLN4	hsa-mir-32-3p	SPAST
hsa-mir-96-5p	LOX	hsa-mir-7-5p	PMEPA1	hsa-mir-32-3p	TCF7L2
hsa-mir-96-5p	MAP3K3	hsa-mir-7-5p	PRDM8	hsa-mir-32-3p	TMF1
hsa-mir-96-5p	MGST1	hsa-mir-7-5p	PARP11	hsa-mir-32-3p	TRPC5
hsa-mir-96-5p	MITF	hsa-mir-7-5p	NIPAL3	hsa-mir-32-3p	VLDLR
hsa-mir-96-5p	PPP1R12A	hsa-mir-7-5p	MIF4GD	hsa-mir-32-3p	YWHAE
hsa-mir-96-5p	NFIC	hsa-mir-7-5p	REXO1	hsa-mir-32-3p	YWHAH
hsa-mir-96-5p	NOTCH2	hsa-mir-7-5p	KIAA1143	hsa-mir-32-3p	MAP3K12
hsa-mir-96-5p	NPTX1	hsa-mir-7-5p	GATAD2B	hsa-mir-32-3p	PTP4A1
hsa-mir-96-5p	PFKFB3	hsa-mir-7-5p	PLEKHH1	hsa-mir-32-3p	FZD6
hsa-mir-96-5p	PFN1	hsa-mir-7-5p	USP31	hsa-mir-32-3p	SNX4
hsa-mir-96-5p	PGAM1	hsa-mir-7-5p	NCEH1	hsa-mir-32-3p	ZRANB2
hsa-mir-96-5p	PGK1	hsa-mir-7-5p	POGK	hsa-mir-32-3p	N4BP1
hsa-mir-96-5p	PMS2	hsa-mir-7-5p	GRAMD1A	hsa-mir-32-3p	DAZAP2
hsa-mir-96-5p	PODXL	hsa-mir-7-5p	CTDSP1	hsa-mir-32-3p	EFCAB14
hsa-mir-96-5p	PON2	hsa-mir-7-5p	PLEKHB1	hsa-mir-32-3p	GPC6
hsa-mir-96-5p	MED1	hsa-mir-7-5p	CACNG7	hsa-mir-32-3p	PPIF
hsa-mir-96-5p	PRKAR1A	hsa-mir-7-5p	SAMSN1	hsa-mir-32-3p	NAMPT
hsa-mir-96-5p	PRKCE	hsa-mir-7-5p	DUS1L	hsa-mir-32-3p	HNRNPR
hsa-mir-96-5p	PTPN9	hsa-mir-7-5p	RBSN	hsa-mir-32-3p	SF3A1
hsa-mir-96-5p	RAB5B	hsa-mir-7-5p	NSD1	hsa-mir-32-3p	SPON2
hsa-mir-96-5p	RAD51	hsa-mir-7-5p	ZNF106	hsa-mir-32-3p	CELF1
hsa-mir-96-5p	RASA1	hsa-mir-7-5p	MPP5	hsa-mir-32-3p	SRSF10
hsa-mir-96-5p	KDM5A	hsa-mir-7-5p	CRTC3	hsa-mir-32-3p	PPARGC1A
hsa-mir-96-5p	RELA	hsa-mir-7-5p	FNDC4	hsa-mir-32-3p	FGFR1OP
hsa-mir-96-5p	RGS2	hsa-mir-7-5p	MARCKSL1	hsa-mir-32-3p	KIF3A
hsa-mir-96-5p	RLF	hsa-mir-7-5p	UBE2Z	hsa-mir-32-3p	PLPBP
hsa-mir-96-5p	RPS23	hsa-mir-7-5p	TRIR	hsa-mir-32-3p	MON1B
hsa-mir-96-5p	RPS29	hsa-mir-7-5p	ZNF655	hsa-mir-32-3p	WDR37
hsa-mir-96-5p	ATXN1	hsa-mir-7-5p	TSEN34	hsa-mir-32-3p	BTBD3
hsa-mir-96-5p	CCL22	hsa-mir-7-5p	ATG9A	hsa-mir-32-3p	ZNF292
hsa-mir-96-5p	SLC1A1	hsa-mir-7-5p	SLC25A23	hsa-mir-32-3p	PEG10
hsa-mir-96-5p	SLC6A6	hsa-mir-7-5p	TMUB2	hsa-mir-32-3p	PPWD1
hsa-mir-96-5p	SNAI2	hsa-mir-7-5p	EFHD2	hsa-mir-32-3p	TMEM2
hsa-mir-96-5p	SOX5	hsa-mir-7-5p	TMEM43	hsa-mir-32-3p	EID1
hsa-mir-96-5p	SUPT4H1	hsa-mir-7-5p	ZNF557	hsa-mir-32-3p	PPIL2
hsa-mir-96-5p	SYCP1	hsa-mir-7-5p	FYCO1	hsa-mir-32-3p	SETBP1
hsa-mir-96-5p	ZEB1	hsa-mir-7-5p	SLC52A2	hsa-mir-32-3p	FAM169A
hsa-mir-96-5p	PPP1R11	hsa-mir-7-5p	RNF128	hsa-mir-32-3p	HBP1
hsa-mir-96-5p	TERF2	hsa-mir-7-5p	RHBDF2	hsa-mir-32-3p	CHORDC1
hsa-mir-96-5p	TIMP1	hsa-mir-7-5p	HECTD3	hsa-mir-32-3p	SIGLEC9
hsa-mir-96-5p	TMPO	hsa-mir-7-5p	ALG9	hsa-mir-32-3p	VPS4A

hsa-mir-96-5p	UBE2N	hsa-mir-7-5p	HPS6	hsa-mir-32-3p	UBE2S
hsa-mir-96-5p	YWHAG	hsa-mir-7-5p	DOCK5	hsa-mir-32-3p	SNX24
hsa-mir-96-5p	ZNF185	hsa-mir-7-5p	NRSN2	hsa-mir-32-3p	GPSM2
hsa-mir-96-5p	ZYX	hsa-mir-7-5p	SEMA6D	hsa-mir-32-3p	HDDC2
hsa-mir-96-5p	EVI5	hsa-mir-7-5p	ATF7IP2	hsa-mir-32-3p	RDH11
hsa-mir-96-5p	DEK	hsa-mir-7-5p	DCAF17	hsa-mir-32-3p	DYNC1LI1
hsa-mir-96-5p	USP5	hsa-mir-7-5p	TMEM134	hsa-mir-32-3p	CYB5R4
hsa-mir-96-5p	RECK	hsa-mir-7-5p	CHD9	hsa-mir-32-3p	RSL24D1
hsa-mir-96-5p	CGGBP1	hsa-mir-7-5p	OPA3	hsa-mir-32-3p	RNF125
hsa-mir-96-5p	B4GALT3	hsa-mir-7-5p	DNAJC5	hsa-mir-32-3p	SBNO1
hsa-mir-96-5p	TNFRSF10A	hsa-mir-7-5p	ZNF436	hsa-mir-32-3p	PPP6R3
hsa-mir-96-5p	MBD4	hsa-mir-7-5p	SETD7	hsa-mir-32-3p	LAPTM4B
hsa-mir-96-5p	SYNGR2	hsa-mir-7-5p	COLEC12	hsa-mir-32-3p	TSR1
hsa-mir-96-5p	ARHGEF2	hsa-mir-7-5p	C1orf21	hsa-mir-32-3p	NAXD
hsa-mir-96-5p	STK17B	hsa-mir-7-5p	TRIM8	hsa-mir-32-3p	TMEM30A
hsa-mir-96-5p	BAG4	hsa-mir-7-5p	TSC22D4	hsa-mir-32-3p	MKL2
hsa-mir-96-5p	EDEM1	hsa-mir-7-5p	GLT8D2	hsa-mir-32-3p	CC2D2A
hsa-mir-96-5p	MTSS1	hsa-mir-7-5p	BCL2L12	hsa-mir-32-3p	USP37
hsa-mir-96-5p	HNRNPDL	hsa-mir-7-5p	C19orf12	hsa-mir-32-3p	KIAA1614
hsa-mir-96-5p	PAK4	hsa-mir-7-5p	ARMC10	hsa-mir-32-3p	PCDHB16
hsa-mir-96-5p	PRMT5	hsa-mir-7-5p	TM2D2	hsa-mir-32-3p	ZNF410
hsa-mir-96-5p	ATG7	hsa-mir-7-5p	STK40	hsa-mir-32-3p	INIP
hsa-mir-96-5p	NUP50	hsa-mir-7-5p	CHCHD5	hsa-mir-32-3p	GPBP1L1
hsa-mir-96-5p	FRS2	hsa-mir-7-5p	LLPH	hsa-mir-32-3p	CIDEC
hsa-mir-96-5p	MALT1	hsa-mir-7-5p	DDI2	hsa-mir-32-3p	RFX7
hsa-mir-96-5p	MORF4L1	hsa-mir-7-5p	MFSD14B	hsa-mir-32-3p	MRPL36
hsa-mir-96-5p	KDELR1	hsa-mir-7-5p	MFSD9	hsa-mir-32-3p	VPS33A
hsa-mir-96-5p	RAB35	hsa-mir-7-5p	LMNB2	hsa-mir-32-3p	WNK1
hsa-mir-96-5p	PLPBP	hsa-mir-7-5p	RIOX2	hsa-mir-32-3p	BBS10
hsa-mir-96-5p	ADNP2	hsa-mir-7-5p	ADO	hsa-mir-32-3p	ATAD5
hsa-mir-96-5p	FBXO21	hsa-mir-7-5p	FAM136A	hsa-mir-32-3p	PHC3
hsa-mir-96-5p	CAMTA1	hsa-mir-7-5p	CORO6	hsa-mir-32-3p	NCALD
hsa-mir-96-5p	SIN3B	hsa-mir-7-5p	MFSD5	hsa-mir-32-3p	MED10
hsa-mir-96-5p	SYNM	hsa-mir-7-5p	RBM17	hsa-mir-32-3p	USMG5
hsa-mir-96-5p	EXOSC2	hsa-mir-7-5p	KRTAP4-2	hsa-mir-32-3p	ZNF587
hsa-mir-96-5p	ABCA6	hsa-mir-7-5p	MICALL1	hsa-mir-32-3p	PPP1R15B
hsa-mir-96-5p	DDAH1	hsa-mir-7-5p	ZIC5	hsa-mir-32-3p	TMEM67
hsa-mir-96-5p	SGK3	hsa-mir-7-5p	CCDC65	hsa-mir-32-3p	COL23A1
hsa-mir-96-5p	TSKU	hsa-mir-7-5p	NAV1	hsa-mir-32-3p	MYADM
hsa-mir-96-5p	MOXD1	hsa-mir-7-5p	RSPRY1	hsa-mir-32-3p	PAXBP1
hsa-mir-96-5p	APPL1	hsa-mir-7-5p	ZNF625	hsa-mir-32-3p	MED12L
hsa-mir-96-5p	PHF19	hsa-mir-7-5p	TRIM47	hsa-mir-32-3p	FAT3
hsa-mir-96-5p	CNNM3	hsa-mir-7-5p	PCED1B	hsa-mir-32-3p	ZNF573
hsa-mir-96-5p	SLC39A1	hsa-mir-7-5p	RPS19BP1	hsa-mir-32-3p	WTIP

hsa-mir-96-5p	TNRC6A	hsa-mir-7-5p	SLFN11	hsa-mir-32-3p	UHMK1
hsa-mir-96-5p	NKIRAS2	hsa-mir-7-5p	SNX29	hsa-mir-32-3p	C5orf24
hsa-mir-96-5p	CDON	hsa-mir-7-5p	TIMM50	hsa-mir-32-3p	NCOA7
hsa-mir-96-5p	TUBD1	hsa-mir-7-5p	FAM114A1	hsa-mir-32-3p	ARL6IP6
hsa-mir-96-5p	POMP	hsa-mir-7-5p	PRDM6	hsa-mir-32-3p	BMT2
hsa-mir-96-5p	SNX7	hsa-mir-7-5p	IGSF8	hsa-mir-32-3p	KIAA1958
hsa-mir-96-5p	REV1	hsa-mir-7-5p	ZFAND4	hsa-mir-32-3p	ZNF567
hsa-mir-96-5p	UBE2D4	hsa-mir-7-5p	TJAP1	hsa-mir-32-3p	CCDC71L
hsa-mir-96-5p	ATP8A2	hsa-mir-7-5p	ERI2	hsa-mir-32-3p	TVP23C
hsa-mir-96-5p	BRWD1	hsa-mir-7-5p	NACC1	hsa-mir-32-3p	TMEM192
hsa-mir-96-5p	CWC25	hsa-mir-7-5p	TEX261	hsa-mir-32-3p	HNRNPA3
hsa-mir-96-5p	ALKBH5	hsa-mir-7-5p	OSBPL8	hsa-mir-32-3p	ZDHHC20
hsa-mir-96-5p	WDR33	hsa-mir-7-5p	OSBPL11	hsa-mir-32-3p	SGMS1
hsa-mir-96-5p	ZC3H15	hsa-mir-7-5p	MAS1L	hsa-mir-32-3p	LINC00346
hsa-mir-96-5p	ASH1L	hsa-mir-7-5p	RFFL	hsa-mir-16-2-3p	AR
hsa-mir-96-5p	ZMIZ1	hsa-mir-7-5p	SP7	hsa-mir-16-2-3p	ATP6V1C1
hsa-mir-96-5p	LRTM1	hsa-mir-7-5p	ACOT4	hsa-mir-16-2-3p	B2M
hsa-mir-96-5p	CNOT6	hsa-mir-7-5p	CANT1	hsa-mir-16-2-3p	BID
hsa-mir-96-5p	TAOK1	hsa-mir-7-5p	LSM12	hsa-mir-16-2-3p	BMI1
hsa-mir-96-5p	CRAMP1	hsa-mir-7-5p	MRPL10	hsa-mir-16-2-3p	CBS
hsa-mir-96-5p	TRIB3	hsa-mir-7-5p	TANGO2	hsa-mir-16-2-3p	CCNT1
hsa-mir-96-5p	PROK2	hsa-mir-7-5p	EMID1	hsa-mir-16-2-3p	CDC25A
hsa-mir-96-5p	PRDM16	hsa-mir-7-5p	TAF8	hsa-mir-16-2-3p	CLCN3
hsa-mir-96-5p	SNX16	hsa-mir-7-5p	ENPP6	hsa-mir-16-2-3p	CTGF
hsa-mir-96-5p	PAPD5	hsa-mir-7-5p	MPLKIP	hsa-mir-16-2-3p	FRK
hsa-mir-96-5p	YTHDC2	hsa-mir-7-5p	UBXN2B	hsa-mir-16-2-3p	HOXA9
hsa-mir-96-5p	GID4	hsa-mir-7-5p	RNF183	hsa-mir-16-2-3p	HOXA10
hsa-mir-96-5p	EFHD2	hsa-mir-7-5p	SPTY2D1	hsa-mir-16-2-3p	IGFBP5
hsa-mir-96-5p	THAP9	hsa-mir-7-5p	KRT80	hsa-mir-16-2-3p	TNPO1
hsa-mir-96-5p	ZFAND1	hsa-mir-7-5p	ZNF555	hsa-mir-16-2-3p	LPP
hsa-mir-96-5p	KLHL15	hsa-mir-7-5p	COMMD7	hsa-mir-16-2-3p	LY75
hsa-mir-96-5p	TSPAN14	hsa-mir-7-5p	DUSP18	hsa-mir-16-2-3p	MYC
hsa-mir-96-5p	PITPNM3	hsa-mir-7-5p	BTLA	hsa-mir-16-2-3p	PRKAA1
hsa-mir-96-5p	SH3BGRL3	hsa-mir-7-5p	RNF38	hsa-mir-16-2-3p	PTPN14
hsa-mir-96-5p	SESN2	hsa-mir-7-5p	C8orf37	hsa-mir-16-2-3p	RAB1A
hsa-mir-96-5p	PPP1R9B	hsa-mir-7-5p	SLC35G1	hsa-mir-16-2-3p	RARB
hsa-mir-96-5p	SPPL2A	hsa-mir-7-5p	SDE2	hsa-mir-16-2-3p	RBBP6
hsa-mir-96-5p	TRIM4	hsa-mir-7-5p	SPTSSB	hsa-mir-16-2-3p	RPS4X
hsa-mir-96-5p	OTULIN	hsa-mir-7-5p	ADAMTS17	hsa-mir-16-2-3p	MLX
hsa-mir-96-5p	SLC25A46	hsa-mir-7-5p	ASXL1	hsa-mir-16-2-3p	TUBB2A
hsa-mir-96-5p	TP53INP1	hsa-mir-7-5p	FAM9C	hsa-mir-16-2-3p	UBE2D3
hsa-mir-96-5p	SMIM12	hsa-mir-7-5p	LDHD	hsa-mir-16-2-3p	VSNL1
hsa-mir-96-5p	SLC25A25	hsa-mir-7-5p	CASP16P	hsa-mir-16-2-3p	YWHAG
hsa-mir-96-5p	GINM1	hsa-mir-7-5p	ALG14	hsa-mir-16-2-3p	ZFX

hsa-mir-96-5p	SLAIN1	hsa-mir-7-5p	SLC39A11	hsa-mir-16-2-3p	CDK2AP1
hsa-mir-96-5p	NIPA1	hsa-mir-7-5p	HTRA4	hsa-mir-16-2-3p	NRIP1
hsa-mir-96-5p	PAQR4	hsa-mir-7-5p	CYB561A3	hsa-mir-16-2-3p	DYRK2
hsa-mir-96-5p	TRIM71	hsa-mir-7-5p	CPNE2	hsa-mir-16-2-3p	AP3B1
hsa-mir-96-5p	IL31RA	hsa-mir-7-5p	UNC5CL	hsa-mir-16-2-3p	ALDH1A2
hsa-mir-96-5p	SPIN4	hsa-mir-7-5p	ANKRD31	hsa-mir-16-2-3p	RPS6KA5
hsa-mir-96-5p	S100A16	hsa-mir-7-5p	BCL9L	hsa-mir-16-2-3p	ABCG2
hsa-mir-96-5p	SRXN1	hsa-mir-7-5p	CHAMP1	hsa-mir-16-2-3p	SH3BP5
hsa-mir-96-5p	MIPOL1	hsa-mir-7-5p	C17orf78	hsa-mir-16-2-3p	EI24
hsa-mir-96-5p	STOML3	hsa-mir-7-5p	FAM126B	hsa-mir-16-2-3p	PREPL
hsa-mir-96-5p	GPR156	hsa-mir-7-5p	C3orf38	hsa-mir-16-2-3p	LRIG2
hsa-mir-96-5p	REEP3	hsa-mir-7-5p	PRRT3	hsa-mir-16-2-3p	CD302
hsa-mir-96-5p	FAM171A1	hsa-mir-7-5p	CAGE1	hsa-mir-16-2-3p	PPIF
hsa-mir-96-5p	TWISTNB	hsa-mir-7-5p	POTED	hsa-mir-16-2-3p	WASF2
hsa-mir-96-5p	JAZF1	hsa-mir-7-5p	KRTAP11-1	hsa-mir-16-2-3p	UBE2E3
hsa-mir-96-5p	RPL7L1	hsa-mir-7-5p	IFNE	hsa-mir-16-2-3p	IPO7
hsa-mir-96-5p	ACER2	hsa-mir-7-5p	CLEC4G	hsa-mir-16-2-3p	CCT4
hsa-mir-96-5p	ATXN1L	hsa-mir-7-5p	GLDN	hsa-mir-16-2-3p	MRPS30
hsa-mir-96-5p	NUP43	hsa-mir-7-5p	KIF24	hsa-mir-16-2-3p	SUB1
hsa-mir-96-5p	MIGA1	hsa-mir-7-5p	LCE1B	hsa-mir-16-2-3p	PAPD7
hsa-mir-96-5p	ANKRD36	hsa-mir-7-5p	LCE1E	hsa-mir-16-2-3p	RNF44
hsa-mir-96-5p	NHLRC3	hsa-mir-7-5p	NCR3LG1	hsa-mir-16-2-3p	ARL6IP1
hsa-mir-96-5p	MTHFD2L	hsa-mir-7-5p	ANKRD36	hsa-mir-16-2-3p	AGO2
hsa-mir-96-5p	CASTOR2	hsa-mir-7-5p	PTAR1	hsa-mir-16-2-3p	SULT1B1
hsa-mir-96-5p	TMEM170B	hsa-mir-7-5p	PNPLA7	hsa-mir-16-2-3p	DROSHA
hsa-mir-96-5p	TRAPPC3L	hsa-mir-7-5p	KMT5A	hsa-mir-16-2-3p	SH3GLB1
hsa-mir-96-5p	ABHD14A-ACY1	hsa-mir-7-5p	TMEM81	hsa-mir-16-2-3p	ERGIC2
hsa-mir-7-5p	ACTC1	hsa-mir-7-5p	ZNF805	hsa-mir-16-2-3p	RSBN1
hsa-mir-7-5p	ACTN2	hsa-mir-7-5p	C10orf105	hsa-mir-16-2-3p	CMTM6
hsa-mir-7-5p	ACVR1B	hsa-mir-7-5p	TMEM236	hsa-mir-16-2-3p	ERBIN
hsa-mir-7-5p	ADCY9	hsa-mir-7-5p	RHOXF2B	hsa-mir-16-2-3p	TM9SF3
hsa-mir-7-5p	PARP1	hsa-mir-7-5p	SIGLEC14	hsa-mir-16-2-3p	NUCKS1
hsa-mir-7-5p	ALDH1A3	hsa-mir-7-5p	KRTAP4-7	hsa-mir-16-2-3p	CCDC14
hsa-mir-7-5p	ALDH3B2	hsa-mir-7-5p	SMIM9	hsa-mir-16-2-3p	TBC1D15
hsa-mir-7-5p	ALDH3A2	hsa-mir-203a-3p	ABL1	hsa-mir-16-2-3p	USP46
hsa-mir-7-5p	ANXA11	hsa-mir-203a-3p	ACVR2B	hsa-mir-16-2-3p	RNF219
hsa-mir-7-5p	XIAP	hsa-mir-203a-3p	AKT2	hsa-mir-16-2-3p	AGMAT
hsa-mir-7-5p	APLP2	hsa-mir-203a-3p	ALOX15	hsa-mir-16-2-3p	KLHL15
hsa-mir-7-5p	ARF4	hsa-mir-203a-3p	BIRC5	hsa-mir-16-2-3p	ANP32E
hsa-mir-7-5p	ARG1	hsa-mir-203a-3p	ASNA1	hsa-mir-16-2-3p	RHNO1
hsa-mir-7-5p	ATP1B3	hsa-mir-203a-3p	ASPA	hsa-mir-16-2-3p	TMEM117
hsa-mir-7-5p	AUP1	hsa-mir-203a-3p	ATM	hsa-mir-16-2-3p	NFATC2IP
hsa-mir-7-5p	BAX	hsa-mir-203a-3p	BCL2L2	hsa-mir-16-2-3p	ACBD5
hsa-mir-7-5p	BCAT1	hsa-mir-203a-3p	BCL7A	hsa-mir-16-2-3p	NACC1

hsa-mir-7-5p	BCL2	hsa-mir-203a-3p	BMI1	hsa-mir-16-2-3p	NUS1
hsa-mir-7-5p	BMPR2	hsa-mir-203a-3p	BMPR1A	hsa-mir-16-2-3p	PABPC4L
hsa-mir-7-5p	CALM3	hsa-mir-203a-3p	BTG1	hsa-mir-16-2-3p	GRPEL2
hsa-mir-7-5p	CALU	hsa-mir-203a-3p	CALR	hsa-mir-16-2-3p	TMEM68
hsa-mir-7-5p	CAMK2D	hsa-mir-203a-3p	CAV1	hsa-mir-16-2-3p	AMOTL1
hsa-mir-7-5p	CANX	hsa-mir-203a-3p	RUNX2	hsa-mir-16-2-3p	ZNF431
hsa-mir-7-5p	CAPN2	hsa-mir-203a-3p	CDH1	hsa-mir-16-2-3p	ZNF585A
hsa-mir-7-5p	CAPZA1	hsa-mir-203a-3p	CDH7	hsa-mir-16-2-3p	PTAR1
hsa-mir-7-5p	CAPZB	hsa-mir-203a-3p	CDK6	hsa-mir-16-2-3p	MTRNR2L6
hsa-mir-7-5p	CASP9	hsa-mir-203a-3p	FOXN3	hsa-mir-16-2-3p	LY75-CD302
hsa-mir-7-5p	CAV1	hsa-mir-203a-3p	CCR5	hsa-mir-16-2-3p	COMMD3-BMI1
hsa-mir-7-5p	CBS	hsa-mir-203a-3p	CREB1	hsa-mir-181c-3p	ADSS
hsa-mir-7-5p	CCNE1	hsa-mir-203a-3p	DLX5	hsa-mir-181c-3p	SERPINC1
hsa-mir-7-5p	CCNT2	hsa-mir-203a-3p	DUSP5	hsa-mir-181c-3p	ATP5G3
hsa-mir-7-5p	SCARB2	hsa-mir-203a-3p	E2F1	hsa-mir-181c-3p	FGF2
hsa-mir-7-5p	ENTPD6	hsa-mir-203a-3p	E2F3	hsa-mir-181c-3p	HOXA9
hsa-mir-7-5p	CDC25B	hsa-mir-203a-3p	EDNRA	hsa-mir-181c-3p	KCNN3
hsa-mir-7-5p	CHRNA2	hsa-mir-203a-3p	EN2	hsa-mir-181c-3p	LIF
hsa-mir-7-5p	ERCC8	hsa-mir-203a-3p	STOM	hsa-mir-181c-3p	RAB5B
hsa-mir-7-5p	CKS2	hsa-mir-203a-3p	EYA4	hsa-mir-181c-3p	SOD2
hsa-mir-7-5p	CLK3	hsa-mir-203a-3p	EXT1	hsa-mir-181c-3p	TPM3
hsa-mir-7-5p	TPP1	hsa-mir-203a-3p	FGF2	hsa-mir-181c-3p	G3BP2
hsa-mir-7-5p	CLN3	hsa-mir-203a-3p	FRK	hsa-mir-181c-3p	HIPK3
hsa-mir-7-5p	CNN3	hsa-mir-203a-3p	FZD2	hsa-mir-181c-3p	TBR1
hsa-mir-7-5p	CRKL	hsa-mir-203a-3p	G6PC	hsa-mir-181c-3p	RAB32
hsa-mir-7-5p	DCTD	hsa-mir-203a-3p	GABRB1	hsa-mir-181c-3p	ADAMTS13
hsa-mir-7-5p	DEFA5	hsa-mir-203a-3p	GAS1	hsa-mir-181c-3p	RAB14
hsa-mir-7-5p	DHCR7	hsa-mir-203a-3p	GATA6	hsa-mir-181c-3p	FAM193B
hsa-mir-7-5p	SLC26A3	hsa-mir-203a-3p	GNAS	hsa-mir-181c-3p	RAB22A
hsa-mir-7-5p	DSC2	hsa-mir-203a-3p	RAPGEF1	hsa-mir-181c-3p	JCAD
hsa-mir-7-5p	DTYMK	hsa-mir-203a-3p	GSK3B	hsa-mir-181c-3p	MMP25
hsa-mir-7-5p	E2F2	hsa-mir-203a-3p	HOXA1	hsa-mir-181c-3p	C16orf58
hsa-mir-7-5p	PHC2	hsa-mir-203a-3p	HOXD3	hsa-mir-181c-3p	PCYOX1L
hsa-mir-7-5p	EGFR	hsa-mir-203a-3p	HTR2A	hsa-mir-181c-3p	C22orf46
hsa-mir-7-5p	EIF2S3	hsa-mir-203a-3p	IFIT1	hsa-mir-181c-3p	TBL1XR1
hsa-mir-7-5p	EIF4E	hsa-mir-203a-3p	IGF1R	hsa-mir-181c-3p	SFXN3
hsa-mir-7-5p	EIF4EBP2	hsa-mir-203a-3p	IGFBP5	hsa-mir-181c-3p	TMEM175
hsa-mir-7-5p	ENSA	hsa-mir-203a-3p	IL6	hsa-mir-181c-3p	MIEF2
hsa-mir-7-5p	FGF1	hsa-mir-203a-3p	IL7	hsa-mir-181c-3p	FAM241A
hsa-mir-7-5p	FLNA	hsa-mir-203a-3p	CXCL8	hsa-mir-181c-3p	TET3
hsa-mir-7-5p	FOS	hsa-mir-203a-3p	TNFRSF9	hsa-mir-181c-3p	GPR137C
hsa-mir-7-5p	GALNT2	hsa-mir-203a-3p	FOXK2	hsa-mir-181c-3p	POTED
hsa-mir-7-5p	GALNT3	hsa-mir-203a-3p	JUN	hsa-mir-181c-3p	C8orf17
hsa-mir-7-5p	GATA6	hsa-mir-203a-3p	KCNJ2	hsa-mir-500a-5p	APP

hsa-mir-7-5p	GDNF	hsa-mir-203a-3p	KIF2A	hsa-mir-500a-5p	SLC25A20
hsa-mir-7-5p	GFI1	hsa-mir-203a-3p	KIF5B	hsa-mir-500a-5p	CCNT1
hsa-mir-7-5p	GFRA1	hsa-mir-203a-3p	LASP1	hsa-mir-500a-5p	CDC5L
hsa-mir-7-5p	GGCX	hsa-mir-203a-3p	LDHA	hsa-mir-500a-5p	CRX
hsa-mir-7-5p	GLO1	hsa-mir-203a-3p	LIFR	hsa-mir-500a-5p	CYLD
hsa-mir-7-5p	GLS	hsa-mir-203a-3p	SMAD2	hsa-mir-500a-5p	EXTL3
hsa-mir-7-5p	GNA12	hsa-mir-203a-3p	SMAD4	hsa-mir-500a-5p	FOXC1
hsa-mir-7-5p	GPR19	hsa-mir-203a-3p	SMAD9	hsa-mir-500a-5p	FSHB
hsa-mir-7-5p	GPR20	hsa-mir-203a-3p	MBNL1	hsa-mir-500a-5p	FUT2
hsa-mir-7-5p	GRB2	hsa-mir-203a-3p	MMP1	hsa-mir-500a-5p	GABRG2
hsa-mir-7-5p	GRIN2D	hsa-mir-203a-3p	MMP10	hsa-mir-500a-5p	GALK2
hsa-mir-7-5p	GRINA	hsa-mir-203a-3p	MYD88	hsa-mir-500a-5p	GAS1
hsa-mir-7-5p	PDIA3	hsa-mir-203a-3p	NARS	hsa-mir-500a-5p	GNAQ
hsa-mir-7-5p	HDLBP	hsa-mir-203a-3p	NCL	hsa-mir-500a-5p	GPM6B
hsa-mir-7-5p	HELLS	hsa-mir-203a-3p	NEK3	hsa-mir-500a-5p	GRIN2B
hsa-mir-7-5p	SLC29A2	hsa-mir-203a-3p	NFYA	hsa-mir-500a-5p	GTF2F2
hsa-mir-7-5p	HNRNPU	hsa-mir-203a-3p	NPPC	hsa-mir-500a-5p	H2AFX
hsa-mir-7-5p	HOXA3	hsa-mir-203a-3p	OSBP	hsa-mir-500a-5p	HAS2
hsa-mir-7-5p	HOXB3	hsa-mir-203a-3p	REG3A	hsa-mir-500a-5p	HSD3B1
hsa-mir-7-5p	HOXB5	hsa-mir-203a-3p	PAX6	hsa-mir-500a-5p	KLRD1
hsa-mir-7-5p	HOXC12	hsa-mir-203a-3p	PDE7A	hsa-mir-500a-5p	MAOA
hsa-mir-7-5p	HPCAL1	hsa-mir-203a-3p	PIK3CA	hsa-mir-500a-5p	MBNL1
hsa-mir-7-5p	HRH2	hsa-mir-203a-3p	PLAGL2	hsa-mir-500a-5p	MCM2
hsa-mir-7-5p	IGSF3	hsa-mir-203a-3p	PLD2	hsa-mir-500a-5p	MAP3K9
hsa-mir-7-5p	IDE	hsa-mir-203a-3p	PPP1CB	hsa-mir-500a-5p	MYO5A
hsa-mir-7-5p	IDH3A	hsa-mir-203a-3p	PRKACB	hsa-mir-500a-5p	PIP4K2A
hsa-mir-7-5p	IGF1R	hsa-mir-203a-3p	PRKCA	hsa-mir-500a-5p	PKNOX1
hsa-mir-7-5p	IGFBP4	hsa-mir-203a-3p	MAPK8	hsa-mir-500a-5p	PRKAA2
hsa-mir-7-5p	IGFBP5	hsa-mir-203a-3p	MAPK9	hsa-mir-500a-5p	PTPN14
hsa-mir-7-5p	RBPJ	hsa-mir-203a-3p	PRNP	hsa-mir-500a-5p	RBBP4
hsa-mir-7-5p	IL12RB2	hsa-mir-203a-3p	RGL2	hsa-mir-500a-5p	REL
hsa-mir-7-5p	FOXK2	hsa-mir-203a-3p	RAN	hsa-mir-500a-5p	RPS4X
hsa-mir-7-5p	ILF2	hsa-mir-203a-3p	RAP1A	hsa-mir-500a-5p	RPS6KB1
hsa-mir-7-5p	ILF3	hsa-mir-203a-3p	RAP2B	hsa-mir-500a-5p	SRSF1
hsa-mir-7-5p	IRS1	hsa-mir-203a-3p	RASA2	hsa-mir-500a-5p	SRSF2
hsa-mir-7-5p	JARID2	hsa-mir-203a-3p	ABCE1	hsa-mir-500a-5p	SLC1A1
hsa-mir-7-5p	KCNH2	hsa-mir-203a-3p	SCO1	hsa-mir-500a-5p	SLC1A2
hsa-mir-7-5p	KCNJ10	hsa-mir-203a-3p	SIX1	hsa-mir-500a-5p	SLC2A3
hsa-mir-7-5p	KCNJ14	hsa-mir-203a-3p	SNAI2	hsa-mir-500a-5p	SLC8A1
hsa-mir-7-5p	KIF5B	hsa-mir-203a-3p	SNAI1	hsa-mir-500a-5p	SNRPD3
hsa-mir-7-5p	KPNB1	hsa-mir-203a-3p	SOD2	hsa-mir-500a-5p	STAT1
hsa-mir-7-5p	KRT7	hsa-mir-203a-3p	SON	hsa-mir-500a-5p	STK4
hsa-mir-7-5p	LAMC2	hsa-mir-203a-3p	SRC	hsa-mir-500a-5p	TACC1
hsa-mir-7-5p	LGALS3BP	hsa-mir-203a-3p	STAT1	hsa-mir-500a-5p	NR2C2

hsa-mir-7-5p	LRP6	hsa-mir-203a-3p	SYK	hsa-mir-500a-5p	ZNF227
hsa-mir-7-5p	MAP1B	hsa-mir-203a-3p	TCF4	hsa-mir-500a-5p	PRRC2A
hsa-mir-7-5p	MAZ	hsa-mir-203a-3p	ZEB1	hsa-mir-500a-5p	DNAH17
hsa-mir-7-5p	MEF2D	hsa-mir-203a-3p	DYNLT1	hsa-mir-500a-5p	UNC5C
hsa-mir-7-5p	ATXN3	hsa-mir-203a-3p	NR2F2	hsa-mir-500a-5p	TAX1BP1
hsa-mir-7-5p	ABCC1	hsa-mir-203a-3p	NKX2-1	hsa-mir-500a-5p	CPNE1
hsa-mir-7-5p	MSH3	hsa-mir-203a-3p	TNF	hsa-mir-500a-5p	MBD4
hsa-mir-7-5p	MYC	hsa-mir-203a-3p	TOP2A	hsa-mir-500a-5p	GCM2
hsa-mir-7-5p	MYLK	hsa-mir-203a-3p	TPD52L1	hsa-mir-500a-5p	MED17
hsa-mir-7-5p	NBL1	hsa-mir-203a-3p	NR2C2	hsa-mir-500a-5p	NPEPPS
hsa-mir-7-5p	NDUFA4	hsa-mir-203a-3p	TRPS1	hsa-mir-500a-5p	RGS6
hsa-mir-7-5p	44806	hsa-mir-203a-3p	TYMS	hsa-mir-500a-5p	CCP110
hsa-mir-7-5p	NFYA	hsa-mir-203a-3p	SUMO1	hsa-mir-500a-5p	UBE4B
hsa-mir-7-5p	NRAS	hsa-mir-203a-3p	UVRAG	hsa-mir-500a-5p	POLR3G
hsa-mir-7-5p	ROR1	hsa-mir-203a-3p	VEGFA	hsa-mir-500a-5p	YME1L1
hsa-mir-7-5p	OAS2	hsa-mir-203a-3p	ZNF24	hsa-mir-500a-5p	KIF1C
hsa-mir-7-5p	PA2G4	hsa-mir-203a-3p	ZNF148	hsa-mir-500a-5p	NUP50
hsa-mir-7-5p	PRDX1	hsa-mir-203a-3p	ZMYM2	hsa-mir-500a-5p	SRSF10
hsa-mir-7-5p	PAK1	hsa-mir-203a-3p	ZNF200	hsa-mir-500a-5p	ZNF460
hsa-mir-7-5p	PAPPA	hsa-mir-203a-3p	MAFK	hsa-mir-500a-5p	MTHFD2
hsa-mir-7-5p	PAX6	hsa-mir-203a-3p	NCOA4	hsa-mir-500a-5p	AAK1
hsa-mir-7-5p	PDE4D	hsa-mir-203a-3p	ANP32A	hsa-mir-500a-5p	KDM1A
hsa-mir-7-5p	PFN2	hsa-mir-203a-3p	ARID1A	hsa-mir-500a-5p	TTLL12
hsa-mir-7-5p	SERPINB5	hsa-mir-203a-3p	PIP5K1A	hsa-mir-500a-5p	SIK3
hsa-mir-7-5p	PIGH	hsa-mir-203a-3p	DYRK3	hsa-mir-500a-5p	NNT
hsa-mir-7-5p	PIK3CB	hsa-mir-203a-3p	CUL3	hsa-mir-500a-5p	TTC33
hsa-mir-7-5p	PIK3CD	hsa-mir-203a-3p	PPM1D	hsa-mir-500a-5p	FRRS1L
hsa-mir-7-5p	PIK3CG	hsa-mir-203a-3p	CASK	hsa-mir-500a-5p	PPIL2
hsa-mir-7-5p	PLAGL2	hsa-mir-203a-3p	TP63	hsa-mir-500a-5p	BAMBI
hsa-mir-7-5p	PLEC	hsa-mir-203a-3p	DDX3Y	hsa-mir-500a-5p	POLR1A
hsa-mir-7-5p	PMP2	hsa-mir-203a-3p	SNX4	hsa-mir-500a-5p	PLEKHG3
hsa-mir-7-5p	POLR2E	hsa-mir-203a-3p	BANF1	hsa-mir-500a-5p	C16orf72
hsa-mir-7-5p	PPP2R1B	hsa-mir-203a-3p	CDKL2	hsa-mir-500a-5p	SLC35B3
hsa-mir-7-5p	MAPK9	hsa-mir-203a-3p	SOCS3	hsa-mir-500a-5p	RMDN1
hsa-mir-7-5p	MAP2K2	hsa-mir-203a-3p	MAP3K13	hsa-mir-500a-5p	COMMD2
hsa-mir-7-5p	THAP12	hsa-mir-203a-3p	SOCS6	hsa-mir-500a-5p	ERGIC2
hsa-mir-7-5p	PTK2	hsa-mir-203a-3p	CIAO1	hsa-mir-500a-5p	MOV10L1
hsa-mir-7-5p	PTK7	hsa-mir-203a-3p	TJP2	hsa-mir-500a-5p	PXK
hsa-mir-7-5p	PURB	hsa-mir-203a-3p	RASAL2	hsa-mir-500a-5p	CDKAL1
hsa-mir-7-5p	RAB3A	hsa-mir-203a-3p	ROCK2	hsa-mir-500a-5p	PLEKHB2
hsa-mir-7-5p	RAF1	hsa-mir-203a-3p	CLOCK	hsa-mir-500a-5p	SYNJ2BP
hsa-mir-7-5p	RALA	hsa-mir-203a-3p	VGLL4	hsa-mir-500a-5p	TMX4
hsa-mir-7-5p	REL	hsa-mir-203a-3p	KIAA0408	hsa-mir-500a-5p	OTUD7B
hsa-mir-7-5p	RELA	hsa-mir-203a-3p	DAZAP2	hsa-mir-500a-5p	CYSLTR2

hsa-mir-7-5p	RPL15	hsa-mir-203a-3p	TSC22D2	hsa-mir-500a-5p	SCYL3
hsa-mir-7-5p	RPS3A	hsa-mir-203a-3p	ZEB2	hsa-mir-500a-5p	VANGL2
hsa-mir-7-5p	RSU1	hsa-mir-203a-3p	HELZ	hsa-mir-500a-5p	VPS18
hsa-mir-7-5p	RYK	hsa-mir-203a-3p	SLC23A1	hsa-mir-500a-5p	INIP
hsa-mir-7-5p	ATXN1	hsa-mir-203a-3p	THRAP3	hsa-mir-500a-5p	NYX
hsa-mir-7-5p	CXCL5	hsa-mir-203a-3p	HNRNPDL	hsa-mir-500a-5p	RAB17
hsa-mir-7-5p	SDHC	hsa-mir-203a-3p	DNAJB6	hsa-mir-500a-5p	C16orf58
hsa-mir-7-5p	SRSF1	hsa-mir-203a-3p	RNF41	hsa-mir-500a-5p	PRRG4
hsa-mir-7-5p	SKP2	hsa-mir-203a-3p	HNRNPR	hsa-mir-500a-5p	NOX5
hsa-mir-7-5p	SLC5A3	hsa-mir-203a-3p	DSCR3	hsa-mir-500a-5p	GSTCD
hsa-mir-7-5p	SLC6A9	hsa-mir-203a-3p	DLC1	hsa-mir-500a-5p	DNAJB14
hsa-mir-7-5p	SLIT3	hsa-mir-203a-3p	IPO7	hsa-mir-500a-5p	MPIG6B
hsa-mir-7-5p	SLPI	hsa-mir-203a-3p	HEXIM1	hsa-mir-500a-5p	GSG1
hsa-mir-7-5p	SMARCD1	hsa-mir-203a-3p	ARID3B	hsa-mir-500a-5p	FSD1L
hsa-mir-7-5p	SNCA	hsa-mir-203a-3p	KHDRBS1	hsa-mir-500a-5p	SLC7A6OS
hsa-mir-7-5p	SOD2	hsa-mir-203a-3p	CELF2	hsa-mir-500a-5p	POLR1B
hsa-mir-7-5p	CAPN15	hsa-mir-203a-3p	KIF1C	hsa-mir-500a-5p	PARD6B
hsa-mir-7-5p	SRM	hsa-mir-203a-3p	NUP50	hsa-mir-500a-5p	PRRC2B
hsa-mir-7-5p	SRPK1	hsa-mir-203a-3p	ARPP19	hsa-mir-500a-5p	FAM104A
hsa-mir-7-5p	SSX1	hsa-mir-203a-3p	ZNF268	hsa-mir-500a-5p	ZIC5
hsa-mir-7-5p	STK11	hsa-mir-203a-3p	IL24	hsa-mir-500a-5p	ANKRD40
hsa-mir-7-5p	SULT1C2	hsa-mir-203a-3p	CIT	hsa-mir-500a-5p	CYP2U1
hsa-mir-7-5p	SUPT4H1	hsa-mir-203a-3p	FGFR1OP	hsa-mir-500a-5p	TUBGCP5
hsa-mir-7-5p	SURF6	hsa-mir-203a-3p	ZWINT	hsa-mir-500a-5p	GGT6
hsa-mir-7-5p	TAF1	hsa-mir-203a-3p	WIF1	hsa-mir-500a-5p	LYPD6
hsa-mir-7-5p	TAF4	hsa-mir-203a-3p	POLI	hsa-mir-500a-5p	DNAJC21
hsa-mir-7-5p	TAGLN	hsa-mir-203a-3p	SEC63	hsa-mir-500a-5p	CKAP2L
hsa-mir-7-5p	TAPBP	hsa-mir-203a-3p	PDCD10	hsa-mir-500a-5p	ITPRIPL1
hsa-mir-7-5p	TCOF1	hsa-mir-203a-3p	PARK7	hsa-mir-500a-5p	NFXL1
hsa-mir-7-5p	PPP1R11	hsa-mir-203a-3p	ZNF652	hsa-mir-500a-5p	SLC38A9
hsa-mir-7-5p	TFPI	hsa-mir-203a-3p	FAM208A	hsa-mir-500a-5p	AMOT
hsa-mir-7-5p	TFRC	hsa-mir-203a-3p	SATB2	hsa-mir-500a-5p	SLC35G1
hsa-mir-7-5p	TGM2	hsa-mir-203a-3p	ZCCHC11	hsa-mir-500a-5p	PGBD4
hsa-mir-7-5p	TLR4	hsa-mir-203a-3p	SYNM	hsa-mir-500a-5p	FAM9C
hsa-mir-7-5p	TNFAIP2	hsa-mir-203a-3p	SMCHD1	hsa-mir-500a-5p	CT62
hsa-mir-7-5p	TNP1	hsa-mir-203a-3p	UFL1	hsa-mir-500a-5p	USF3
hsa-mir-7-5p	TOP2A	hsa-mir-203a-3p	SLC44A1	hsa-mir-500a-5p	SLC36A1
hsa-mir-7-5p	TSN	hsa-mir-203a-3p	SH3BP4	hsa-mir-500a-5p	OR9Q1
hsa-mir-7-5p	UBE2A	hsa-mir-203a-3p	HECTD1	hsa-mir-500a-5p	ZDHHC20
hsa-mir-7-5p	NR1H2	hsa-mir-203a-3p	ZNF451	hsa-mir-500a-5p	ST6GALNAC3
hsa-mir-7-5p	VDAC1	hsa-mir-203a-3p	SZRD1	hsa-mir-500a-5p	DCAF4L1
hsa-mir-7-5p	WFS1	hsa-mir-203a-3p	FBXL3	hsa-mir-500a-5p	SREK1IP1
hsa-mir-7-5p	WNT8B	hsa-mir-203a-3p	FBXL5	hsa-mir-500a-5p	FFAR4
hsa-mir-7-5p	XRCC2	hsa-mir-203a-3p	CNNM4	hsa-mir-500a-5p	OTOG

hsa-mir-7-5p	XRCC5	hsa-mir-203a-3p	CNNM3	hsa-mir-500a-5p	KCNT2
hsa-mir-7-5p	YY1	hsa-mir-203a-3p	RGS17	hsa-mir-500a-5p	FAM71F2
hsa-mir-7-5p	ZP3	hsa-mir-203a-3p	GREM1	hsa-mir-500a-5p	SFT2D2
hsa-mir-7-5p	ZXDA	hsa-mir-203a-3p	TMEM97	hsa-mir-500a-5p	BLOC1S3
hsa-mir-7-5p	ZYX	hsa-mir-203a-3p	TBK1	hsa-mir-500a-5p	SRRD
hsa-mir-7-5p	LUZP1	hsa-mir-203a-3p	SERTAD3	hsa-mir-500a-5p	ZBTB34
hsa-mir-7-5p	DNALI1	hsa-mir-203a-3p	PSAT1	hsa-mir-500a-5p	DENND6B
hsa-mir-7-5p	BTG2	hsa-mir-203a-3p	DNTTIP2	hsa-mir-500a-5p	C8orf82
hsa-mir-7-5p	CUL5	hsa-mir-203a-3p	ASAP1	hsa-mir-500a-5p	RNF165
hsa-mir-7-5p	PTP4A2	hsa-mir-203a-3p	RNF141	hsa-mir-500a-5p	FAM229B
hsa-mir-7-5p	SSPN	hsa-mir-203a-3p	GLRX2	hsa-mir-500a-5p	ZBTB8B
hsa-mir-7-5p	FXR1	hsa-mir-203a-3p	TRNT1	hsa-mir-500a-5p	TMEM242
hsa-mir-7-5p	GAN	hsa-mir-203a-3p	SH3GLB1	hsa-mir-550a-5p	CBS
hsa-mir-7-5p	SLC7A5	hsa-mir-203a-3p	RLIM	hsa-mir-550a-5p	KLF6
hsa-mir-7-5p	AKAP1	hsa-mir-203a-3p	GDAP1	hsa-mir-550a-5p	CPM
hsa-mir-7-5p	GDF5	hsa-mir-203a-3p	EGLN1	hsa-mir-550a-5p	DHODH
hsa-mir-7-5p	OR1A1	hsa-mir-203a-3p	KLHL28	hsa-mir-550a-5p	DYRK1A
hsa-mir-7-5p	RGS5	hsa-mir-203a-3p	NSD3	hsa-mir-550a-5p	EHHADH
hsa-mir-7-5p	PIK3R3	hsa-mir-203a-3p	TMEM70	hsa-mir-550a-5p	ERCC1
hsa-mir-7-5p	YARS	hsa-mir-203a-3p	DNAAF2	hsa-mir-550a-5p	GABPB1
hsa-mir-7-5p	AKR7A2	hsa-mir-203a-3p	ZNF654	hsa-mir-550a-5p	GALNT2
hsa-mir-7-5p	RDH16	hsa-mir-203a-3p	FBXW7	hsa-mir-550a-5p	B4GALT1
hsa-mir-7-5p	PLPP3	hsa-mir-203a-3p	PI4K2B	hsa-mir-550a-5p	KCNJ11
hsa-mir-7-5p	IRS2	hsa-mir-203a-3p	SLC39A9	hsa-mir-550a-5p	MKI67
hsa-mir-7-5p	VAMP8	hsa-mir-203a-3p	TSR1	hsa-mir-550a-5p	COX1
hsa-mir-7-5p	GBF1	hsa-mir-203a-3p	ENAH	hsa-mir-550a-5p	NINJ1
hsa-mir-7-5p	GPAA1	hsa-mir-203a-3p	EXOC2	hsa-mir-550a-5p	NPR1
hsa-mir-7-5p	FADD	hsa-mir-203a-3p	CAND1	hsa-mir-550a-5p	PCCB
hsa-mir-7-5p	SOCS2	hsa-mir-203a-3p	C20orf24	hsa-mir-550a-5p	RAB13
hsa-mir-7-5p	CFLAR	hsa-mir-203a-3p	GPCPD1	hsa-mir-550a-5p	RAD51
hsa-mir-7-5p	ST3GAL5	hsa-mir-203a-3p	DHX33	hsa-mir-550a-5p	RBMS2
hsa-mir-7-5p	SQSTM1	hsa-mir-203a-3p	CDC42SE2	hsa-mir-550a-5p	RPS6KB1
hsa-mir-7-5p	SGPL1	hsa-mir-203a-3p	SLC45A4	hsa-mir-550a-5p	SNAPC3
hsa-mir-7-5p	AP1M1	hsa-mir-203a-3p	RAB22A	hsa-mir-550a-5p	SOX11
hsa-mir-7-5p	TRPA1	hsa-mir-203a-3p	CYP20A1	hsa-mir-550a-5p	ZNF70
hsa-mir-7-5p	HAP1	hsa-mir-203a-3p	SLC12A5	hsa-mir-550a-5p	ZNF154
hsa-mir-7-5p	UNC119	hsa-mir-203a-3p	SERINC1	hsa-mir-550a-5p	SMC1A
hsa-mir-7-5p	XPR1	hsa-mir-203a-3p	SRGAP1	hsa-mir-550a-5p	FZD6
hsa-mir-7-5p	ZBTB22	hsa-mir-203a-3p	NUFIP2	hsa-mir-550a-5p	EIF3F
hsa-mir-7-5p	KLF4	hsa-mir-203a-3p	TRMT5	hsa-mir-550a-5p	STX16
hsa-mir-7-5p	NREP	hsa-mir-203a-3p	HOMEZ	hsa-mir-550a-5p	APLN
hsa-mir-7-5p	GTF3C5	hsa-mir-203a-3p	SLAIN2	hsa-mir-550a-5p	FOXH1
hsa-mir-7-5p	CNOT8	hsa-mir-203a-3p	KIF13A	hsa-mir-550a-5p	LIMD1
hsa-mir-7-5p	SNAP29	hsa-mir-203a-3p	ARHGEF28	hsa-mir-550a-5p	KCNK6

hsa-mir-7-5p	ZNF264	hsa-mir-203a-3p	SMURF2	hsa-mir-550a-5p	TMEM59
hsa-mir-7-5p	LITAF	hsa-mir-203a-3p	RMND5A	hsa-mir-550a-5p	KLHL21
hsa-mir-7-5p	MPDU1	hsa-mir-203a-3p	BCL11B	hsa-mir-550a-5p	HNRNPDL
hsa-mir-7-5p	VPS26A	hsa-mir-203a-3p	RAPH1	hsa-mir-550a-5p	ACTR1A
hsa-mir-7-5p	TCL1B	hsa-mir-203a-3p	WDR77	hsa-mir-550a-5p	DCAF7
hsa-mir-7-5p	SLC25A44	hsa-mir-203a-3p	FYCO1	hsa-mir-550a-5p	BPNT1
hsa-mir-7-5p	UBE3C	hsa-mir-203a-3p	MCTP1	hsa-mir-550a-5p	KIF1C
hsa-mir-7-5p	TRAM2	hsa-mir-203a-3p	CBLL1	hsa-mir-550a-5p	ZNF277
hsa-mir-7-5p	SECISBP2L	hsa-mir-203a-3p	KLHL15	hsa-mir-550a-5p	NT5C2
hsa-mir-7-5p	SUSD6	hsa-mir-203a-3p	CPEB4	hsa-mir-550a-5p	TTC28
hsa-mir-7-5p	JADE3	hsa-mir-203a-3p	CXXC4	hsa-mir-550a-5p	HAUS5
hsa-mir-7-5p	MATR3	hsa-mir-203a-3p	CLPTM1L	hsa-mir-550a-5p	EXOSC2
hsa-mir-7-5p	SERTAD2	hsa-mir-203a-3p	NETO2	hsa-mir-550a-5p	MOB4
hsa-mir-7-5p	DAZAP2	hsa-mir-203a-3p	MRO	hsa-mir-550a-5p	UBXN7
hsa-mir-7-5p	KEAP1	hsa-mir-203a-3p	NCALD	hsa-mir-550a-5p	TMX2
hsa-mir-7-5p	SPATA2	hsa-mir-203a-3p	PCGF6	hsa-mir-550a-5p	GDE1
hsa-mir-7-5p	TRIM14	hsa-mir-203a-3p	POLR1B	hsa-mir-550a-5p	MYO3A
hsa-mir-7-5p	GINS1	hsa-mir-203a-3p	GINS4	hsa-mir-550a-5p	ELP6
hsa-mir-7-5p	AKT3	hsa-mir-203a-3p	ZBED3	hsa-mir-550a-5p	RNF43
hsa-mir-7-5p	GJC1	hsa-mir-203a-3p	C15orf48	hsa-mir-550a-5p	SEMA4C
hsa-mir-7-5p	SCAMP2	hsa-mir-203a-3p	LCOR	hsa-mir-550a-5p	SLC38A7
hsa-mir-7-5p	PPIF	hsa-mir-203a-3p	PARD6B	hsa-mir-550a-5p	YOD1
hsa-mir-7-5p	WASF2	hsa-mir-203a-3p	DGAT2	hsa-mir-550a-5p	NKRF
hsa-mir-7-5p	SLC25A15	hsa-mir-203a-3p	NUDCD1	hsa-mir-550a-5p	CLK4
hsa-mir-7-5p	RNF41	hsa-mir-203a-3p	TRIM4	hsa-mir-550a-5p	STRIP2
hsa-mir-7-5p	ALG3	hsa-mir-203a-3p	MIDN	hsa-mir-550a-5p	KIAA1328
hsa-mir-7-5p	PSME3	hsa-mir-203a-3p	MCFD2	hsa-mir-550a-5p	WNK1
hsa-mir-7-5p	MFSD10	hsa-mir-203a-3p	FMNL2	hsa-mir-550a-5p	NKAP
hsa-mir-7-5p	DCAF7	hsa-mir-203a-3p	FLYWCH2	hsa-mir-550a-5p	ZNF556
hsa-mir-7-5p	SIGMAR1	hsa-mir-203a-3p	44623	hsa-mir-550a-5p	MED28
hsa-mir-7-5p	BCKDK	hsa-mir-203a-3p	COX20	hsa-mir-550a-5p	KLHL15
hsa-mir-7-5p	LANCL1	hsa-mir-203a-3p	GSTO2	hsa-mir-550a-5p	CPEB4
hsa-mir-7-5p	HMGN4	hsa-mir-203a-3p	NAA30	hsa-mir-550a-5p	CDT1
hsa-mir-7-5p	CRTAP	hsa-mir-203a-3p	MSI2	hsa-mir-550a-5p	CDCA7
hsa-mir-7-5p	FBLN5	hsa-mir-203a-3p	ANKRD13B	hsa-mir-550a-5p	QRFPR
hsa-mir-7-5p	IPO7	hsa-mir-203a-3p	CCSAP	hsa-mir-550a-5p	ZNF394
hsa-mir-7-5p	AGPAT1	hsa-mir-203a-3p	TRIM71	hsa-mir-550a-5p	ABRAXAS1
hsa-mir-7-5p	TGOLN2	hsa-mir-203a-3p	SPATA18	hsa-mir-550a-5p	ZDHHC18
hsa-mir-7-5p	PNMA2	hsa-mir-203a-3p	JMY	hsa-mir-550a-5p	PPP1R15B
hsa-mir-7-5p	ZNF460	hsa-mir-203a-3p	PM20D2	hsa-mir-550a-5p	YTHDC1
hsa-mir-7-5p	FRS2	hsa-mir-203a-3p	MTPN	hsa-mir-550a-5p	NT5C1B
hsa-mir-7-5p	ZNF275	hsa-mir-203a-3p	TOR1AIP2	hsa-mir-550a-5p	ZNF561
hsa-mir-7-5p	EHD1	hsa-mir-203a-3p	UBXN2A	hsa-mir-550a-5p	HSPA12B
hsa-mir-7-5p	TMED2	hsa-mir-203a-3p	GPR156	hsa-mir-550a-5p	ISCA2

hsa-mir-7-5p	CKAP4	hsa-mir-203a-3p	PRICKLE2	hsa-mir-550a-5p	SNX20
hsa-mir-7-5p	TMED10	hsa-mir-203a-3p	GLIS3	hsa-mir-550a-5p	PABPC4L
hsa-mir-7-5p	RAB32	hsa-mir-203a-3p	OIT3	hsa-mir-550a-5p	LSM11
hsa-mir-7-5p	CDC37	hsa-mir-203a-3p	ZNF367	hsa-mir-550a-5p	FMC1
hsa-mir-7-5p	WDR45	hsa-mir-203a-3p	ARID2	hsa-mir-550a-5p	UNC5B
hsa-mir-7-5p	FICD	hsa-mir-203a-3p	TRIML2	hsa-mir-550a-5p	FAM102B
hsa-mir-7-5p	AKAP11	hsa-mir-203a-3p	TBCEL	hsa-mir-550a-5p	YIPF6
hsa-mir-7-5p	CHP1	hsa-mir-203a-3p	SPATA13	hsa-mir-550a-5p	ZDHHC21
hsa-mir-7-5p	LYPLA2	hsa-mir-203a-3p	ZUFSP	hsa-mir-550a-5p	ANKRD62
hsa-mir-7-5p	TUSC2	hsa-mir-203a-3p	C6orf223	hsa-mir-550a-5p	RD3
hsa-mir-7-5p	EXOC3	hsa-mir-203a-3p	FOXK1	hsa-mir-550a-5p	FRRS1
hsa-mir-7-5p	MGLL	hsa-mir-203a-3p	SEMA3D	hsa-mir-550a-5p	TMEM151B
hsa-mir-7-5p	RRAS2	hsa-mir-203a-3p	PRR14L	hsa-mir-550a-5p	RNF222
hsa-mir-7-5p	MRAS	hsa-mir-203a-3p	IPMK	hsa-mir-550a-5p	PCP4L1
hsa-mir-7-5p	ATF5	hsa-mir-203a-3p	LCLAT1	hsa-mir-550a-5p	ZBTB8B
hsa-mir-7-5p	ZNF507	hsa-mir-203a-3p	CADM2	hsa-mir-550a-5p	ZNF878
hsa-mir-7-5p	ZNF365	hsa-mir-203a-3p	MCM9	hsa-mir-550a-5p	PSAPL1
hsa-mir-7-5p	SIRT2	hsa-mir-203a-3p	GXYLT1	hsa-mir-550a-5p	NT5C1B-RDH14
hsa-mir-7-5p	MYH15	hsa-mir-203a-3p	HEPHL1	hsa-mir-550a-5p	HSPE1-MOB4
hsa-mir-7-5p	SETD1B	hsa-mir-203a-3p	LCE1A	hsa-mir-942-3p	ALDH1A3
hsa-mir-7-5p	PPRC1	hsa-mir-203a-3p	PEAR1	hsa-mir-942-3p	APBB2
hsa-mir-7-5p	TBC1D2B	hsa-mir-203a-3p	CERKL	hsa-mir-942-3p	ZFP36L2
hsa-mir-7-5p	TNRC6B	hsa-mir-203a-3p	BOLA3	hsa-mir-942-3p	CD44
hsa-mir-7-5p	RAD54L2	hsa-mir-203a-3p	LIN28B	hsa-mir-942-3p	CREBL2
hsa-mir-7-5p	TTLL12	hsa-mir-203a-3p	PRAMEF8	hsa-mir-942-3p	CTH
hsa-mir-7-5p	ZCCHC14	hsa-mir-203a-3p	PIM3	hsa-mir-942-3p	GAS1
hsa-mir-7-5p	ZC3H4	hsa-mir-203a-3p	MXRA7	hsa-mir-942-3p	GATA6
hsa-mir-7-5p	RPRD2	hsa-mir-203a-3p	PRAMEF7	hsa-mir-942-3p	GCH1
hsa-mir-7-5p	PPIP5K2	hsa-mir-203a-3p	LOH12CR2	hsa-mir-942-3p	HNRNPL
hsa-mir-7-5p	ICOSLG	hsa-mir-203a-3p	ZNF704	hsa-mir-942-3p	HNRNPU
hsa-mir-7-5p	ARHGEF12	hsa-mir-203a-3p	TMPPE	hsa-mir-942-3p	IL7R
hsa-mir-7-5p	SRGAP2	hsa-mir-203a-3p	LINC00598	hsa-mir-942-3p	LDHA
hsa-mir-7-5p	NUDCD3	hsa-mir-203a-3p	STMP1	hsa-mir-942-3p	MMP9
hsa-mir-7-5p	EXOSC2	hsa-mir-203a-3p	GXYLT2	hsa-mir-942-3p	NCL
hsa-mir-7-5p	CBX7	hsa-mir-323a-3p	AADAC	hsa-mir-942-3p	PGGT1B
hsa-mir-7-5p	SNAPIN	hsa-mir-323a-3p	CANX	hsa-mir-942-3p	PLK1
hsa-mir-7-5p	CDC42EP4	hsa-mir-323a-3p	CBR1	hsa-mir-942-3p	PTK6
hsa-mir-7-5p	RUSC1	hsa-mir-323a-3p	CDKN1B	hsa-mir-942-3p	RBMS2
hsa-mir-7-5p	FAM89B	hsa-mir-323a-3p	CHEK1	hsa-mir-942-3p	SCO1
hsa-mir-7-5p	KPNA6	hsa-mir-323a-3p	EREG	hsa-mir-942-3p	SRPK1
hsa-mir-7-5p	HSPBP1	hsa-mir-323a-3p	F5	hsa-mir-942-3p	UBE2A
hsa-mir-7-5p	SH3BP4	hsa-mir-323a-3p	GALNT1	hsa-mir-942-3p	VEGFA
hsa-mir-7-5p	DCAF12	hsa-mir-323a-3p	HNRNPA1	hsa-mir-942-3p	UBL4A
hsa-mir-7-5p	TPGS2	hsa-mir-323a-3p	STMN1	hsa-mir-942-3p	SERF1A

hsa-mir-7-5p	ZNF385A	hsa-mir-323a-3p	SMAD2	hsa-mir-942-3p	ARHGEF2
hsa-mir-7-5p	PNISR	hsa-mir-323a-3p	SMAD3	hsa-mir-942-3p	SH3PXD2A
hsa-mir-7-5p	TSKU	hsa-mir-323a-3p	MAP3K1	hsa-mir-942-3p	SETD1A
hsa-mir-7-5p	TMEM98	hsa-mir-323a-3p	MYC	hsa-mir-942-3p	G3BP1
hsa-mir-7-5p	CNTNAP2	hsa-mir-323a-3p	P2RY11	hsa-mir-942-3p	KLF2
hsa-mir-7-5p	RAB11FIP5	hsa-mir-323a-3p	PLAG1	hsa-mir-942-3p	ZNF460
hsa-mir-7-5p	SZRD1	hsa-mir-323a-3p	PMAIP1	hsa-mir-942-3p	LILRA2
hsa-mir-7-5p	PITPNC1	hsa-mir-323a-3p	PPP1CB	hsa-mir-942-3p	DSTN
hsa-mir-7-5p	LHX6	hsa-mir-323a-3p	PPP1CC	hsa-mir-942-3p	RNF44
hsa-mir-7-5p	CNNM4	hsa-mir-323a-3p	PRKAR1A	hsa-mir-942-3p	C1GALT1C1
hsa-mir-7-5p	MYEOV	hsa-mir-323a-3p	STAT3	hsa-mir-942-3p	PURG
hsa-mir-7-5p	CHORDC1	hsa-mir-323a-3p	TCP1	hsa-mir-942-3p	RMDN1
hsa-mir-7-5p	AP3M1	hsa-mir-323a-3p	TRPS1	hsa-mir-942-3p	ARL6IP4
hsa-mir-7-5p	RNF11	hsa-mir-323a-3p	TSNAX	hsa-mir-942-3p	C21orf62-AS1
hsa-mir-7-5p	LYPD3	hsa-mir-323a-3p	CNBP	hsa-mir-942-3p	QPCTL
hsa-mir-7-5p	CACNG5	hsa-mir-323a-3p	ZXDA	hsa-mir-942-3p	SLC48A1
hsa-mir-7-5p	EIF2AK1	hsa-mir-323a-3p	PLA2G7	hsa-mir-942-3p	LMBR1L
hsa-mir-7-5p	INTU	hsa-mir-323a-3p	SEMA7A	hsa-mir-942-3p	NUFIP2
hsa-mir-7-5p	VPS4A	hsa-mir-323a-3p	UNC5C	hsa-mir-942-3p	TAOK1
hsa-mir-7-5p	SERP1	hsa-mir-323a-3p	TNFRSF10B	hsa-mir-942-3p	CRAMP1
hsa-mir-7-5p	LSM3	hsa-mir-323a-3p	WASL	hsa-mir-942-3p	BEND3
hsa-mir-7-5p	SULT1B1	hsa-mir-323a-3p	GPR50	hsa-mir-942-3p	FAM160B1
hsa-mir-7-5p	CRCP	hsa-mir-323a-3p	SOCS5	hsa-mir-942-3p	AFAP1
hsa-mir-7-5p	RBMS3	hsa-mir-323a-3p	BZW1	hsa-mir-942-3p	SUSD1
hsa-mir-7-5p	MOCS3	hsa-mir-323a-3p	FAM20B	hsa-mir-942-3p	CLPB
hsa-mir-7-5p	TNRC6A	hsa-mir-323a-3p	HMGXB4	hsa-mir-942-3p	RAB1B
hsa-mir-7-5p	ANGPTL3	hsa-mir-323a-3p	TSPAN3	hsa-mir-942-3p	ZBTB45
hsa-mir-7-5p	TMEM97	hsa-mir-323a-3p	LHFPL6	hsa-mir-942-3p	TMEM250
hsa-mir-7-5p	GIT1	hsa-mir-323a-3p	TXNIP	hsa-mir-942-3p	SFXN1
hsa-mir-7-5p	DBNL	hsa-mir-323a-3p	PRDM4	hsa-mir-942-3p	STX1B
hsa-mir-7-5p	SSU72	hsa-mir-323a-3p	FNDC3A	hsa-mir-942-3p	ZNF618
hsa-mir-7-5p	UHRF1	hsa-mir-323a-3p	CLCC1	hsa-mir-942-3p	SLC16A10
hsa-mir-7-5p	SERTAD3	hsa-mir-323a-3p	NUP205	hsa-mir-942-3p	EXOSC6
hsa-mir-7-5p	DNTTIP2	hsa-mir-323a-3p	RAB3GAP2	hsa-mir-942-3p	FOXR2
hsa-mir-7-5p	EHD3	hsa-mir-323a-3p	PNISR	hsa-mir-942-3p	EXOC8
hsa-mir-7-5p	IL21R	hsa-mir-323a-3p	SLC17A5	hsa-mir-942-3p	CCDC50
hsa-mir-7-5p	CRIM1	hsa-mir-323a-3p	PELP1	hsa-mir-942-3p	TOR1AIP2
hsa-mir-7-5p	SHISA5	hsa-mir-323a-3p	MYLIP	hsa-mir-942-3p	CASP16P
hsa-mir-7-5p	TMEM69	hsa-mir-323a-3p	GPSM2	hsa-mir-942-3p	RPL22L1
hsa-mir-7-5p	DNAJC27	hsa-mir-323a-3p	SLC35C2	hsa-mir-942-3p	NUDT7
hsa-mir-7-5p	ZDHHC3	hsa-mir-323a-3p	UTP18	hsa-mir-942-3p	POTEG
hsa-mir-7-5p	SLC25A37	hsa-mir-323a-3p	RMDN1	hsa-mir-942-3p	POTEM
hsa-mir-7-5p	MS4A4A	hsa-mir-323a-3p	INO80D	hsa-mir-942-3p	LINC00598
hsa-mir-7-5p	EIF3L	hsa-mir-323a-3p	CDKN2AIP	hsa-mir-942-3p	SERF1B

hsa-mir-7-5p	RWDD1	hsa-mir-323a-3p	DEPDC1
